# Supplementary material for: Hydride formation pressures and kinetics in individual Pd nanoparticles with systematically varied levels of plastic deformation
Source: Nat Commun. 2025 Oct 17;16:9242. doi: 10.1038/s41467-025-64311-3 (PMC12534434; doi:10.1038/s41467-025-64311-3)
Supplement: Supplementary file 1 — Supplementary Information [file 41467_2025_64311_MOESM1_ESM.pdf]

Supporting Information

for

# Hydrogen Absorption Kinetics and Hydride Formation Pressures in Individual Pd Nanoparticles with Systematically Varied Levels of Plastic Deformation

*Carl Andersson[1], Jonathan Zimmerman[2], Joachim Fritzsche[1], Eugen Rabkin[2,§] and Christoph Langhammer[1*]*

[1]Department of Physics, Chalmers University of Technology; SE-412 96 Gothenburg, Sweden

[2]Department of Materials Science and Engineering, Technion - Israel Institute of Technology, 3200003 Haifa, Israel

Corresponding authors: [§]erabkin@technion.ac.il; [*]clangham@chalmers.se

## 1. SEM images before and after 19 hydrogenation cycles

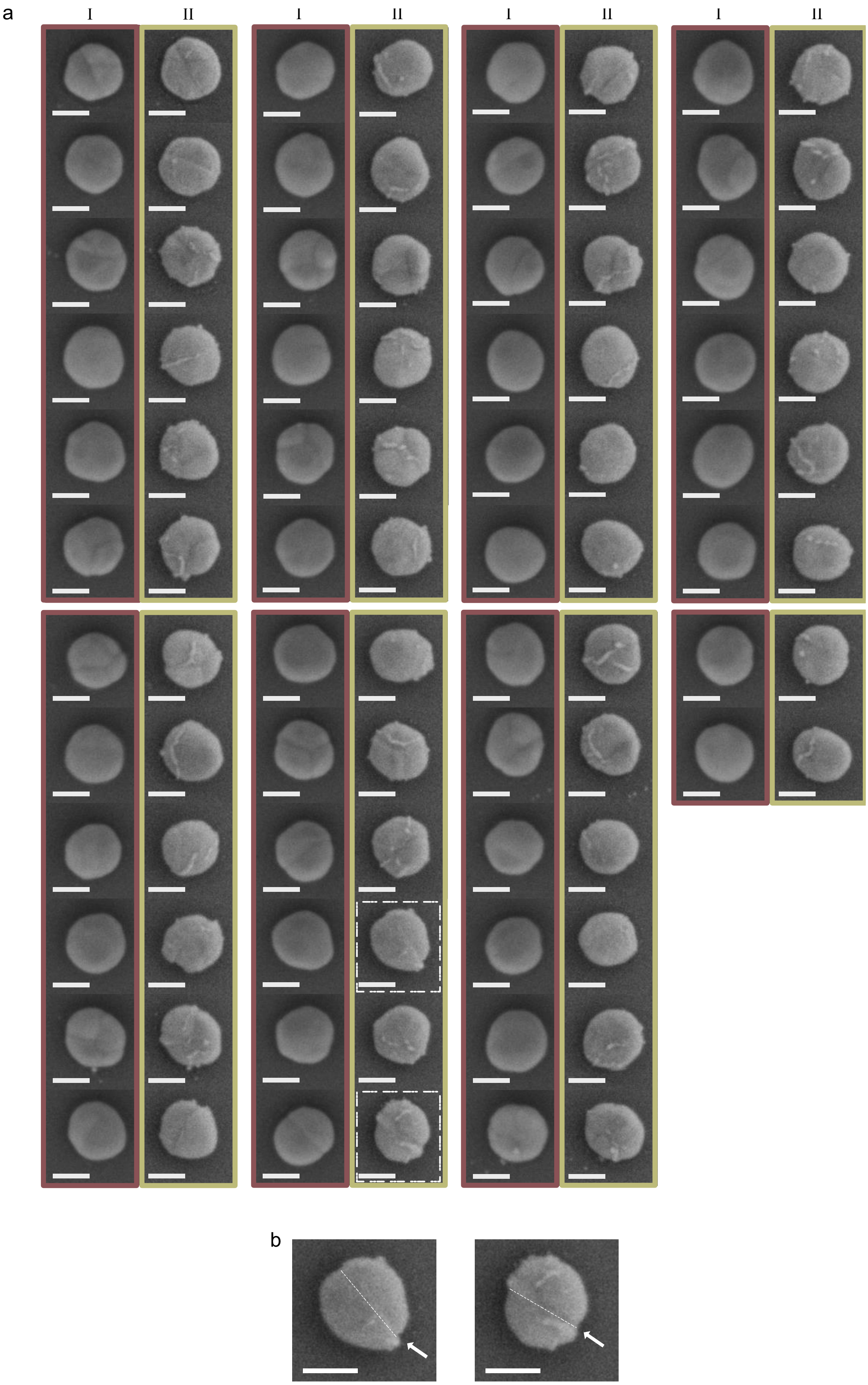
a
I
II
b

**Supplementary Figure 1.** *(a) SEM micrographs of individual Pd nanoparticles before (columns outlined with red boxes and "I" above each column) and after (columns outlined with yellow boxes and "II" above each column) hydrogen cycling. The imaged sample (S2) was fabricated in the same way as the sample discussed in the main text (S1) - except that no particles have been compressed. The particles have been cycled with 19 hydrogenation cycles using the same hydrogenation procedure as for the sample discussed in the main text (S1), see Supplementary Data 1. We note the clearly visible Pd protrusions that have formed on all the particles after cycling. (b) High-magnification SEM micrographs of the particles highlighted with white dashed boxes in (a). Noticeable shear damage to the particles is highlighted by a white arrow and a dashed white line. Scale bars are 100 nm.*

## 2. Load-displacement data of particles in the main text

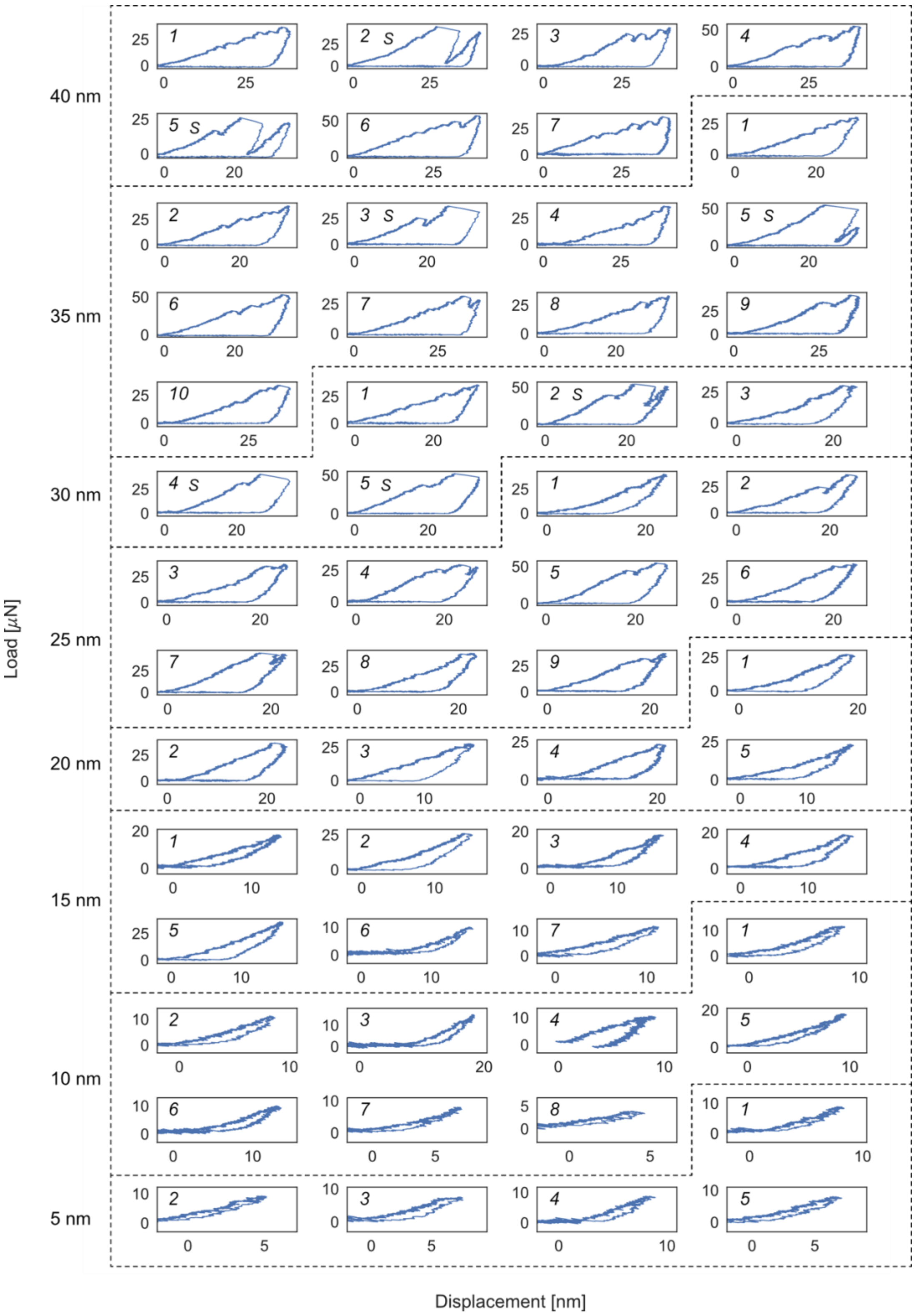


**Supplementary Figure 2.** *Load-displacement diagrams for all 56 compressed particles. The diagrams are numbered in line with* ***Supplementary Figure 19****. Particles experiencing a strain-burst typical for single crystals are marked with an "S".*

| | Particle no | Particle row | Particle column | Displacement nominal [nm] | Displacement real [nm] | Max load [microN] | Strain burst? |
|---|---|---|---|---|---|---|---|
| 40 nm | 1 | 1 | 7 | 40 | 40 | 35 | No |
| | 2 | 1 | 5 | 40 | 45 | 45 | Yes |
| | 3 | 1 | 4 | 40 | 40 | 30 | No |
| | 4 | 1 | 3 | 40 | 45 | 55 | No |
| | 5 | 1 | 2 | 40 | 40 | 25 | Yes |
| | 6 | 1 | 1 | 40 | 40 | 55 | No |
| | 7 | 1 | 10 | 35 | 40 | 35 | No |
| 35 nm | 1 | 1 | 6 | 40 | 35 | 30 | No |
| | 2 | 1 | 14 | 35 | 35 | 35 | No |
| | 3 | 1 | 13 | 35 | 35 | 35 | Yes |
| | 4 | 1 | 12 | 35 | 35 | 35 | No |
| | 5 | 1 | 11 | 35 | 35 | 55 | Yes |
| | 6 | 1 | 9 | 35 | 35 | 50 | No |
| | 7 | 1 | 8 | 35 | 35 | 30 | No |
| | 8 | 1 | 21 | 30 | 35 | 35 | No |
| | 9 | 1 | 17 | 30 | 35 | 40 | No |
| | 10 | 1 | 16 | 30 | 35 | 35 | No |
| 30 nm | 1 | 1 | 20 | 30 | 30 | 35 | No |
| | 2 | 1 | 18 | 30 | 30 | 55 | Yes |
| | 3 | 1 | 15 | 30 | 30 | 30 | No |
| | 4 | 1 | 23 | 25 | 30 | 40 | Yes |
| | 5 | 2 | 3 | 25 | 30 | 50 | Yes |
| 25 nm | 1 | 1 | 19 | 30 | 25 | 40 | No |
| | 2 | 1 | 22 | 25 | 25 | 35 | No |
| | 3 | 2 | 5 | 25 | 25 | 40 | No |
| | 4 | 2 | 4 | 25 | 25 | 30 | No |
| | 5 | 2 | 1 | 25 | 25 | 55 | No |
| | 6 | 2 | 10 | 20 | 25 | 40 | No |
| | 7 | 2 | 9 | 20 | 25 | 45 | No |
| | 8 | 2 | 8 | 20 | 25 | 40 | No |
| | 9 | 2 | 6 | 20 | 25 | 35 | No |
| 20 nm | 1 | 2 | 2 | 25 | 20 | 30 | No |
| | 2 | 2 | 12 | 20 | 20 | 35 | No |
| | 3 | 2 | 11 | 20 | 20 | 30 | No |
| | 4 | 2 | 7 | 20 | 20 | 25 | No |
| | 5 | 2 | 15 | 15 | 20 | 25 | No |
| 15 nm | 1 | 2 | 19 | 15 | 15 | 20 | No |
| | 2 | 2 | 18 | 15 | 15 | 25 | No |
| | 3 | 2 | 17 | 15 | 15 | 20 | No |
| | 4 | 2 | 16 | 15 | 15 | 20 | No |
| | 5 | 2 | 14 | 15 | 15 | 35 | No |
| | 6 | 2 | 13 | 15 | 15 | 10 | No |
| | 7 | 2 | 20 | 10 | 15 | 10 | No |
| 10 nm | 1 | 2 | 23 | 10 | 10 | 10 | No |
| | 2 | 2 | 22 | 10 | 10 | 10 | No |
| | 3 | 2 | 21 | 10 | 10 | 15 | No |
| | 4 | 3 | 3 | 10 | 10 | 10 | No |
| | 5 | 3 | 2 | 10 | 10 | 20 | No |
| | 6 | 3 | 1 | 10 | 10 | 10 | No |
| | 7 | 3 | 9 | 5 | 10 | 10 | No |
| | 8 | 3 | 4 | 5 | 10 | 5 | No |
| 5 nm | 1 | 3 | 10 | 5 | 5 | 10 | No |
| | 2 | 3 | 8 | 5 | 5 | 10 | No |
| | 3 | 3 | 7 | 5 | 5 | 10 | No |
| | 4 | 3 | 6 | 5 | 5 | 10 | No |
| | 5 | 3 | 5 | 5 | 5 | 10 | No |

**Supplementary Figure 3.** *Deformation properties for all nano-compressed particles. Particles are sorted (top to bottom) after their actual measured displacement (i.e. not after nominal displacement values), with "particle no" in accordance with the number on each individual load-displacement diagram in* ***Supplementary Figure 2*** *(and consequently, the same numbering as in* ***Supplementary Figure 19****). Particle column numbering is left-to-right, top-to-bottom of the compressed particles in*

***Figure 2b** of the main text. The true, as well as the target displacement values are presented together with the maximum load that was put on the particle during the compression. The "strain burst" column describes if a particle's deformation was characteristic of a single crystal, as in **Figure 1j** of main text.*

# 3. Estimation of dislocation density post-annealing

Since our particles exhibit a high variation in yield stress, likely due to a high variation in initial dislocation densities, we estimate the latter by studying the upper and lower limit of the compressive strength of our particles. As an upper limit for the load, $F_{crit}$, we use the maximum load applied to any single crystalline "strain burst" particle (55 µN). The lower limit, we estimate from the lowest, clearly visible "step yield" of stair-case yielding particles (~7.5 µN). To calculate the top area of the particles, A, we use the average diameter (196 nm) from the uncompressed particles in **Supplementary Figure 18b**. Note that this diameter technically is measured after the hydrogenation cycling process, but it's close enough to the nominal diameter of the particles (200 nm) that it is still valid. Taken together, the calculated compressive strength $\tau_c = \frac{F_{crit}}{A}$, yields an upper and lower limit of 0.5 and 3.8 GPa respectively.

Next, we apply equation 1 from El-Awady[1]

$$\frac{\tau}{G} = \frac{\beta}{d\sqrt{\rho}} + \alpha b\sqrt{\rho} \tag{1}$$

where $G$ is the shear modulus of Pd (44 GPa), $d$ is the particle diameter, $\rho$ is the dislocation density, $b$ is the Burgers vector (3.89e-10 $\cdot \frac{\sqrt{3}}{2}$ for 111 in Pd) and $\alpha$ and $\beta$ are dimensionless constants from El-Awady[1]. We should here emphasize that depending on how the particles deform, different Burgers vectors could be relevant (the growth direction of our particles is [111], i.e. that we have 111 planes on the top facet of our particles). However, the exact Burgers vector used here for the Pd system is not significantly affecting the outcome of this estimate. Using eq. (1) and solving numerically for $\rho$ gives dislocation densities either on the order $10^{11}$-$10^{12}$ m$^{-2}$ or $10^{14}$-$10^{16}$ m$^{-2}$ (**Supplementary Figure 4a**). Scaling the normalized yield strength $\frac{\tau}{G}$ with $\sqrt{d}$ and the dislocation density $\rho$ with $d$ (**Supplementary Figure 4b**), reproduces the generalized size-dependent crystal strength plot of El-Awady[1]. Here, we note that our minimum limit of the critical load (7.5 µN) results in dislocation densities close to the critical density $\rho_c$, which is the inflection point between the dislocation starvation and forest hardening regime. In fact, if we lower the minimum critical load to 3.4 µN instead of 7.5 µN – which is not an unreasonable assumption for a subset of the particles due to the high scatter of yield strengths – then the two dislocation density estimates (**Supplementary Figure 5**), one on the dislocation starvation flank and one on the forest hardening flank, overlap at the critical dislocation density $\rho_c$, i.e. the dislocation density of many of our particles are likely quite close to $\rho_c$.

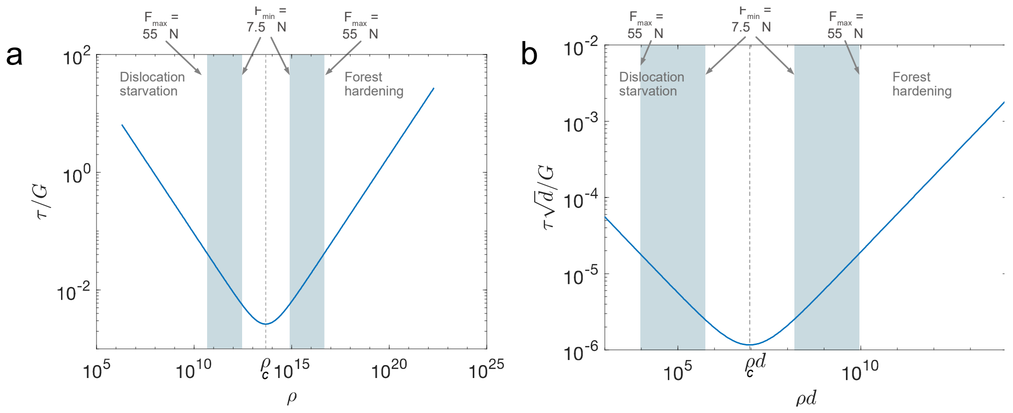


**Supplementary Figure 4.** *Dislocation density estimation. (a) Normalized yield strength as a function of dislocation density $\rho$ using eq. 1. The blue shaded regions are the two regions of $\rho$ that represent the yield strengths of our particles, one region of $\rho \sim 10^{11}$-$10^{12}$ $m^{-2}$ in the dislocation starvation regime (left of the critical dislocation density $\rho_c$), and one region of $\rho \sim 10^{14}$-$10^{16}$ $m^{-2}$ in the forest hardening regime (right of the critical dislocation density $\rho_c$). (b) Scaled normalized yield strength $\tau\sqrt{d}/G$ vs scaled dislocation density $\rho d$*[1].

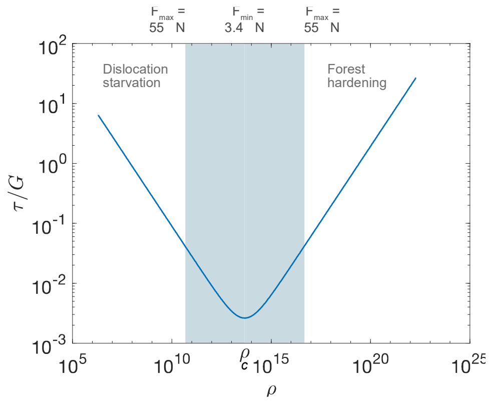


**Supplementary Figure 5.** *Dislocation density calculation using extreme lower limit. Using 3.4 μN instead of 7.5 μN as the limit for the lower yield load results in the two dislocation density estimates (shaded blue regions), one on the dislocation starvation flank and one on the forest hardening flank, to overlap at the critical dislocation density $\rho_c$.*

Taken together, this tells us that we have a significant spread in initial dislocation densities in our particles, and as such a high variation in yield stress, with many particles having dislocation densities close to the critical dislocation density $\rho_c$. Consequently, we can't from this analysis alone say whether the subgroup of particles with higher yield stress are in the dislocation starvation regime or the forest hardening regime. However, if we compare this to recent studies on softening of Pd particles[2], where the hydrogen cycled (soft) particles had a dislocation density around $10^{14}$ $m^{-2}$ (which consequently is around the value of $\rho_c$ for this size of particles), we argue that its likely our particles have initial dislocation densities (i.e. after annealing but before hydrogen cycling) lower than for these cycled particles. Also, as stated in section 4 below, dislocation densities in the range $10^{14}$-$10^{16}$ $m^{-2}$ are expected in the compressed particles. Taken together, the likely initial dislocation densities for our particles are in the $10^{11}$-$10^{12}$ $m^{-2}$ range rather than the $10^{14}$-$10^{16}$ $m^{-2}$ range, which would put our non-deformed particles in the dislocation starvation regime where it is feasible for pre-existing dislocations to glide to and annihilate at the surface at increased stress levels, e.g. during hydrogenation.

## 4. Estimation of post-compression dislocation density

Atomistic molecular dynamics (MD) simulations from the literature can provide an estimate of dislocation density in the deformed nanoparticles. Such simulations have been performed for the faceted single crystalline nanoparticles of several pure metals with face centered cubic (FCC) structure, such as Au[3], Ni[4], Pt[5], and Cu[6], and also for compression of rounded Pd particles[7]. The conclusions of these works are similar – the dislocation nucleation is a stochastic process, so that even two neighboring particles of identical dimensions deformed to the same compressive strain may exhibit different dislocation substructures. In the case of Cu particles, the dislocation densities in the compressed particles derived from MD simulations were about $2\times10^{15}$ m$^{-2}$ in the range of strains of 0.05-0.30, and $1.5\times10^{16}$ m$^{-2}$ in the range of strains of 0.30-0.65. The densities of geometrically necessary dislocations in compressed Pt particles determined experimentally employing electron backscattering diffraction mapping were about $1\times10^{15}$ m$^{-2}$ in the range of strains of 0.05-0.40, and $4\times10^{15}$ m$^{-2}$ in the range of strains of 0.40-0.80[8]. As can be seen, both atomistic MD simulations and experimental studies give the same order of magnitude of dislocation density in deformed FCC nanoparticles in the range of $10^{15}$–$10^{16}$ m$^{-2}$, depending on plastic strain (the plastic strains in this study are on the order of 0.083-0.667, representative of a 5 and 40 nm compression respectively), and also demonstrate high scatter of these values.

## 5. Experimental setup

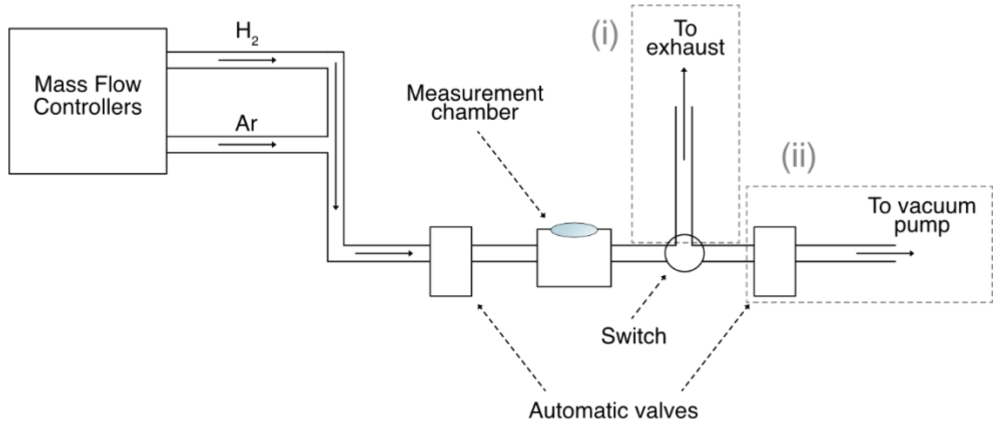


**Supplementary Figure 6.** *Schematic of the experimental setup. The samples are placed inside a gas-tight measurement chamber with optical access through a top-mounted glass window. The chamber has a feedback-controlled heating stage to keep the sample at a stable temperature. An inlet and outlet provide gas exchange through automatic valves. Mass flow controllers provide controllable gas flows that are mixed in the inlet piping. An automatic valve controls when the gas is introduced into the measurement chamber. A switch point provides the choice between i) continuous flow isothermal experiments where the gas is fed to the ventilation exhaust or ii) a second automatic valve which leads to a vacuum pump, which is used for kinetics experiments in vacuum.*

## 6. Oxide reduction and the effect of other potential contaminants

The faster hydrogenation kinetics of the nanocompressed particles could at first glance be explained as a result of the nanocompression process simply removing or "cracking open" the oxide shell that would be expected to exist to some degree on the surface of the newly fabricated Pd nanoparticles. However,

three arguments speak against why oxides or other surface contaminants should affect our findings in any significant way. (i) Hydrogenation of metals generally, and specifically for Pd already at low temperatures, reduce surface oxides[9,10], so after a few cycles the particles should be in a relatively pristine surface state. Indeed, the particle kinetics speed up over the first hydrogenation cycles (see **Figure 2c-e** and cycle 1-5 of **Figure 3a** of the main text) to thereafter plateau (cycle 6-18 of **Figure 3a** of the main text) and therefore corroborates the argument of surface oxide reduction. After these initial cycles, all particles – both compressed and non-compressed – have comparable absorption times (see **Figure 2e** and cycle 5 - 10 in **Figure 3a** of the main text). The main deacceleration of the non-compressed particles happens after this stage (after cycle 18, see **Figure 2f** and **Figure 3a** of the main text). For this deacceleration to be the result of some detrimental surface contaminants, it would require (ii) these contaminants to be present on the particles beforehand or (iii) the contaminants are introduced during the measurement. Here we remind ourselves that all particles in the main text, compressed as well as non-compressed, are located on the same sample and are measured simultaneously. For point (ii) and (iii) regarding surface contaminants from above, this means the following. If contaminants would be present on the sample already from the fabrication, and be the reason for the drastic deacceleration of the non-compressed particles according to point (ii), this would require these contaminants to suddenly "activate" on the non-compressed particles after around 18 cycles – which we deem highly unlikely. If contaminants on the other hand would be introduced during the measurements according to point (iii), this would mean that said contaminants mainly attach to and affect the non-compressed particles – which we also deem highly unlikely. To further corroborate this point, it is clear from the spatial distribution of the particle absorption times for a late hydrogenation cycle (**Supplementary Figure 7**) that there are no localized "slow" areas of non-compressed particles. With all this taken together, we argue that the effect of oxides or other surface contaminants would not be significant enough to take away from the main findings of this paper.

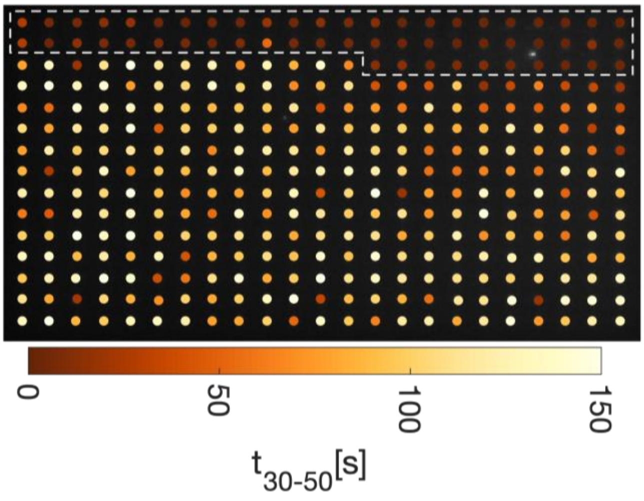


**Supplementary Figure 7.** *Spatial distribution of particles with their corresponding $t_{30-50}$ absorption times at hydrogenation cycle 69. The nanocompressed particles are highlighted with a white dashed box. We note that among the non-compressed particles, there are no isolated, slow outlier areas, i.e. the different absorption times are evenly distributed.*

## 7. Pressure-composition isotherm principle

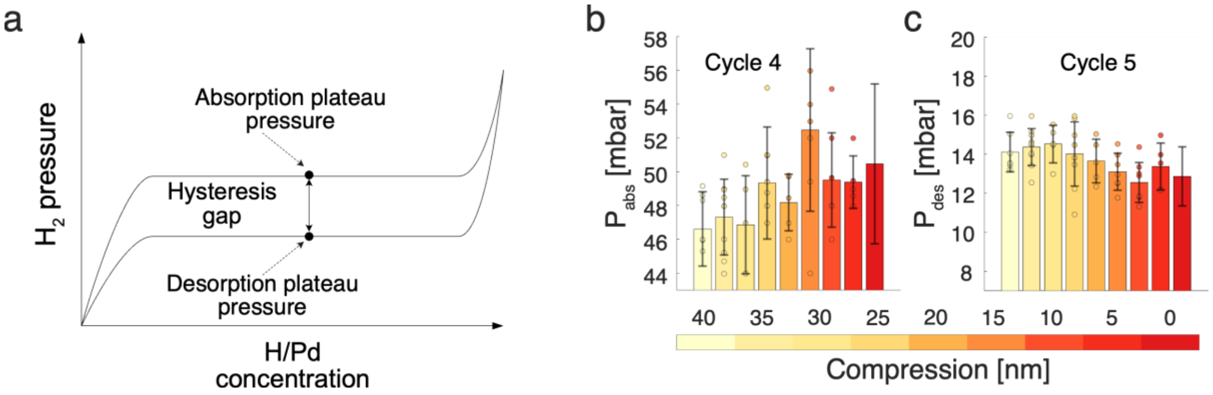


**Supplementary Figure 8.** *(a) Schematic of an idealized hydrogen pressure-composition isotherm for Pd. The plateau pressures for the α-to-β phase transformation during absorption (and vice versa for desorption) defines the hysteresis gap between the absorption and desorption branch of the hydrogenation process. (b) Mean absorption ($P_{abs}$) and desorption ($P_{des}$) plateau pressures for all particles in the 9 different compression-level sub-groups during the first isotherm measurements (cycle 4 and 5 respectively). The error bars indicate one standard deviation calculated from the individual plateau pressures of the particles within each compression group. For the compressed particles, the individual data points are also plotted as filled circles.*

## 8. Kinetic and isotherm data vs total time spent in hydrogenated state

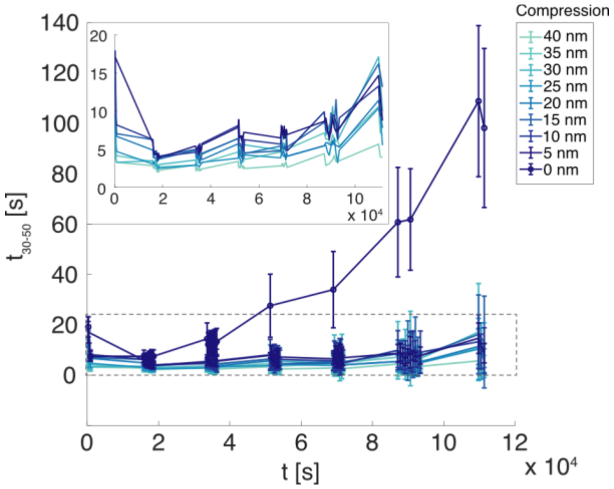


**Supplementary Figure 9.** *Evolution of the average $t_{30-50}$ absorption times for the 9 compression-level sub-groups as a function of total time spent in > 40 mbar $H_2$. The error bars indicate one standard deviation calculated from the individual $t_{30-50}$ absorption times of the particles within each compression-level sub-group. We note the extreme deacceleration of the kinetics for the non-compressed particles (0 nm compression) in later cycles. Inset: Magnification of the average absorption times for the 5 – 40 nm compressed particles (area outlined with a dashed, gray box in the main figure).*

## 9. Absorption time scaling law for Pd single crystals

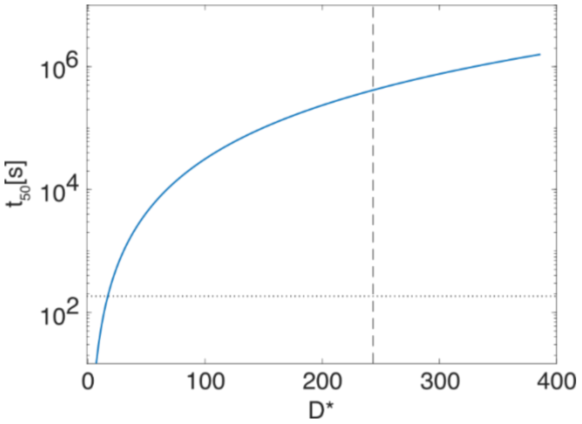


**Supplementary Figure 10.** *Absorption time scaling law for Pd single crystals as a function of effective size extrapolated from Langhammer et al.[11]. The $t_{50}$ absorption time is defined as the time to reach 50 % of the signal change during an optical hydrogenation measurement of Pd particles. The effective size D* is calculated from an equal-volume-sphere based on the volume of our (nominally) disc-shaped nanoparticles (dashed, vertical line) and the $t_{5\text{-}50}$ absorption time (calculated as the time between 5 % and 50 % signal change) for the undeformed particles during cycle 69 is presented as dotted, horizontal line.*

## 10. Method to extract kinetics data only from particles that have fully desorbed

When hydrogenation kinetics measurements are performed, gas is pumped out of the test chamber for a certain amount of time (typically 20-30 min) before the next hydrogen pulse is introduced. However, if the desorption kinetics become very slow, this usual pumping duration might not be enough to let the particles completely desorb before the next cycle starts. To illustrate this, two particles - one compressed and one non-compressed – are presented in **Supplementary Figure 11** during two later cycles (cycle 59 and 60). The compressed particle (**Supplementary Figure 11a**) have completely desorbed between the first and second hydrogen pulse. This leads to the drops in the signal intensity due to the hydrogen absorption ($\Delta I_1$ and $\Delta I_2 = 0.95\, \Delta I_1$), being comparable in magnitude to each other. The non-compressed particle on the other hand (**Supplementary Figure 11b**) does not completely desorb between the two consecutive hydrogen pulses, and as such the drops in the intensity signal $\Delta I_1$ and $\Delta I_2 = 0.70\, \Delta I_1$) are not close to each other in magnitude, leading to erroneous $t_{30\text{-}50}$ values if comparing the two cycles for this particle (as the two $t_{30\text{-}50}$ absorption times will represent different parts of the absorption process). Therefore, in all plots that show any type of average of the kinetic evolution for the non-compressed particles (**Figure 3a** and **Figure 5d** of the main text as well as **Supplementary Figure 9**, **Supplementary Figure 15**, **Supplementary Figure 23** and **Supplementary Figure 29e-f**), a condition was imposed that at least 200 of the 289 non-compressed particles in every cycle should have intensity drops within 90 % of the first cycle in that kinetics measurement set. A “kinetics measurement set” is defined here as all cycles between isothermal cycles (i.e. all cycles in between dashed lines of **Figure 3a** of the main text). As the first cycle in every kinetics measurement set is directly following an isothermal desorption measurement (where a particle spends hours desorbing), the particles can be more or less guaranteed to have fully desorbed before these cycles. If a cycle failed to reach the condition stated above, that cycle was discarded.

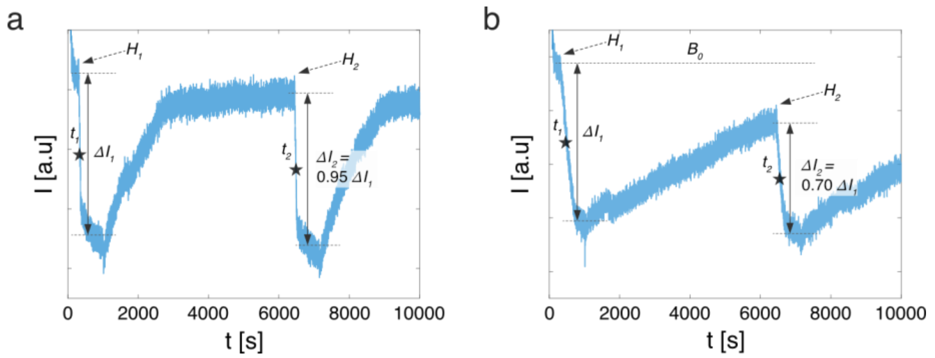


**Supplementary Figure 11.** *Intensity evolution of two different, one compressed (a) and one non-compressed (b), particles during two consecutive hydrogen cycles (cycle 59 and 60) from a kinetics measurement. Hydrogen introduction events for every cycle is marked with an H, and the intensity drop resulting from each introduction event (due to the α-to-β phase transformation) is marked with $\Delta I_1$ (first cycle) and $\Delta I_2$ (second cycle) respectively. The half-point of each intensity drop is marked with a black star for each cycle and the time for the half-point (with respect to the hydrogen introduction events $H_1$ and $H_2$) are $t_1$ and $t_2$ respectively. Note that in b, the intensity is far from reaching the baseline $B_0$ before the second hydrogen pulse is introduced ($H_2$). This means that the particle in question has not completely desorbed, and therefore the absorption time $t_2$ will be erroneous.*

## 11. The influence of slow kinetics on isothermal measurements

When measuring the Pd phase transition plateau pressures with pressure-composition isotherms, it is inherently assumed that enough time is given at every hydrogen pressure step for the particles to equilibrate to the current hydrogen pressure before a new, either higher (absorption isotherm) or lower (desorption isotherm), pressure is introduced. However, if the particle's sorption kinetics slow down significantly, this may affect the pressure-composition isotherms and push the sorption plateau pressures to higher (lower) apparent plateau pressures for absorption (desorption), *e.g.* **Supplementary Figure 12**. To ensure comparability between the compressed and non-compressed particles, which exhibit significantly different absorption times after about cycle 30 (**Figure 3a** of the main text), only particles with absorption times comparable to the compressed particles ($t_{30\text{-}50}$ < 25 s) were included in the plots showing the evolution of the absorption plateau pressures $P_{abs}$ (**Figure 3b** of the main text, **Supplementary Figure 13**, **Supplementary Figure 14a-b**, **Supplementary Figure 17** and **Supplementary Figure 30a-b**). This problem is the most apparent for the intermediate cycles, i.e. after cycle 30 (where absorption times for the non-compressed particles start to significantly diverge from the compressed particles, **Figure 3a** of the main text) and before cycle 75 (where the time per hydrogen pressure step was significantly increased from 300 s per step to 1200 s and 2400 s per step for absorption and desorption respectively). Therefore, the absorption plateau pressures ($P_{abs}$) for all non-compressed particles are used in **Supplementary Figure 24b-g** and **Supplementary Figure 30c-d** as the cycles shown there are outside the intermediate region of cycles.

For the individual absorption plateau pressures ($P_{abs}$) of all non-compressed particles during all absorption isotherms together with their corresponding absorption times, see **Supplementary Figure 27**.

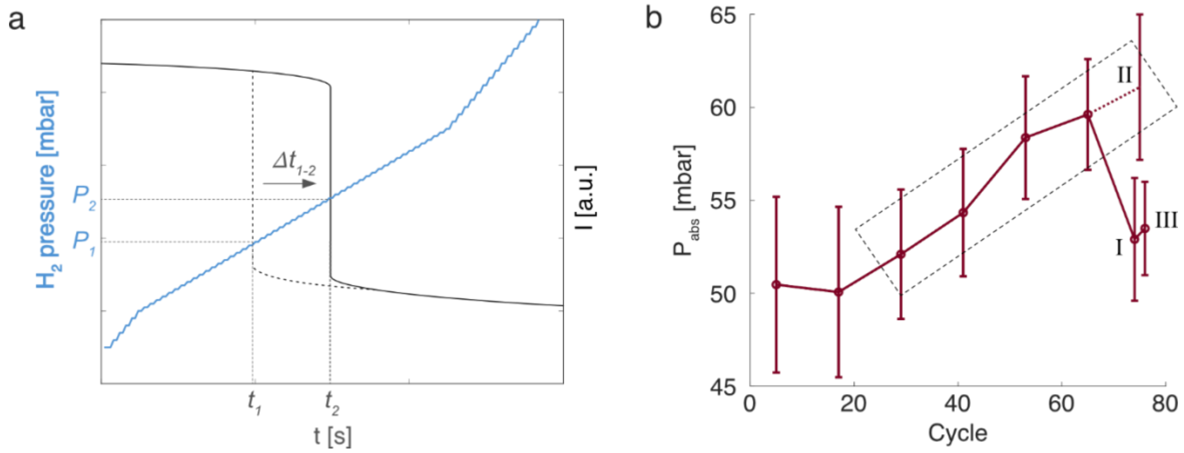


**Supplementary Figure 12.** *(a) Schematic representation of slow absorption kinetics influencing the apparent hydrogen absorption plateau pressure of a Pd nanoparticle. The phase change from the α- to the β-phase of the Pd-hydrogen system is resolved through the back-scattered intensity (black line) of the Pd nanoparticle as a sharp drop.[12] If we assume that this phase change would occur at hydrogen pressure $P_1$, we should measure the intensity drop around time $t_1$. However, if the particle absorbs the hydrogen very slowly (compared to the time of one step in hydrogen pressure), the intensity drop can be delayed by a time $\Delta t_{1-2}$ to time $t_2$, which would give a higher, apparent phase change pressure of $P_2$. (b) Evolution of the average absorption ($P_{abs}$) plateau pressures for all non-compressed particles as a function of hydrogenation cycles when taking all measured isotherms into account. Two of the last three isotherms (I and III) were measured with a 1200 s step per 1 mbar $H_2$ step and the isotherm in between (II) was measured with a 300 s step per 1 mbar $H_2$ step. The distinct increase to higher $P_{abs}$ pressures when using the shorter step time (II) indicates that the rise in absorption plateau pressures seen for the non-compressed particles during the intermediate isotherm measurements (dashed box) may be apparent and instead a result of the significant deacceleration of absorption kinetics seen for the non-compressed particles during the corresponding cycles in* ***Figure 3a*** *of the main text. The error bars indicate one standard deviation calculated from the individual plateau pressures of every particle.*

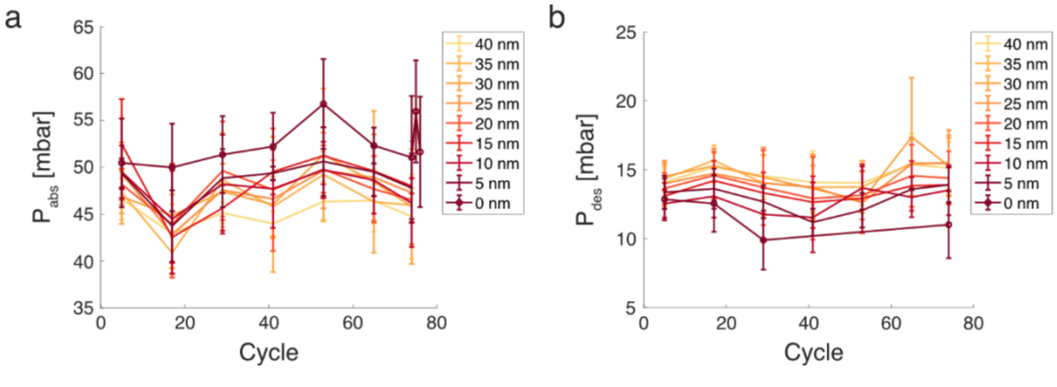


**Supplementary Figure 13.** *The evolution of the average absorption (a) and desorption (b) plateau pressures for the 9 different compression-level sub-groups groups as a function of hydrogenation cycles. The error bars indicate one standard deviation calculated from the individual plateau pressures of the particles within each compression group. For the non-compressed (0 nm) particles in a, only particles with absorption times comparable to the compressed particles ($t_{30-50}$< 25 s) were included and for b, the intermediate cycles (75 > cycle > 30) were left out in accordance with the discussion of the main text in this section.*

## 12. The evolution of isothermal plateau pressures – expanded

The evolution of the isothermal plateau pressures goes through five distinct phases during the hydrogen cycling process (**Figure 3b** of the main text and **Supplementary Figure 14a-b**). The first phase (I) is characterized by a decrease in absorption pressure ($P_{abs}$) for all particle groups (**Supplementary Figure 14a-b**). According to the Schwarz-Khachaturyan-Griessen model, this would imply a decrease of strain levels during hydrogenation during phase I – but this model technically only holds for defect-free particles. This means that for the two compressed sub-groups (5-20 nm and 25-40 nm compression), the decrease in $P_{abs}$ could also be an effect of, as is stated in the main text, the dislocations generated during the nanocompression acting as nucleation sources for the β-phase hydride and thereby reducing the thermodynamic nucleation barrier of the hydride phase. However, we will assume in the rest of this section that the Schwarz-Khachaturyan-Griessen model (SKG-model), and as such that $P_{abs}$ is directly related to the overall strain state of the particles, holds for the non-compressed (0 nm compression) particles. As such, this would dictate that the strain levels of the non-compressed particles develop as follows.

During phase I, the strain levels of the particles are decreasing (average $P_{abs}$ is decreasing), most likely due to a crystal restructuring mediated by the clamping stress of the substrate during hydrogenation (for more information, see the main text as well as **Supplementary Figure 1** and **Supplementary Figure 20**), where Pd atoms diffuse from the highly strained interfacial region towards the less strained surface region.

In phase II, the average $P_{abs}$ increase, which according to the SKG-model would imply an increase in strain levels. To the best of our knowledge, there could be two different origins for these increased stress levels during hydrogenation of the particles, i) migration and subsequent annihilation of existing dislocations at grain boundaries or the surface of the crystal, leading the particles to becoming more and more defect-free and thereby raising $P_{abs}$[13], or ii) new dislocations form during the hydrogenation process to such a degree a that a version of strain hardening arises in the particles, and thereby $P_{abs}$ is increased due to the higher $H_2$ pressure needed to induce the α-to-β phase transition. However, since phase II coincide with the cycles where the non-compressed particles start to deaccelerate significantly (see **Figure 3a** in the main text), the argument of dislocation movement and annihilation seems the most likely.

In phase III, the average $P_{abs}$ is again decreasing, which would imply a second release of stress from the particles, potentially from further diffusion of Pd away from the clamped surface or by the formation of new stress releasing dislocations. We find the latter of these mechanisms, i.e. the formation of new dislocations, to be the most plausible in this scenario, especially since it directly follows the suggested dislocation annihilation of phase II. As such, the particles go through a cyclic dislocation formation-annihilation over ~50 cycles of hydrogen cycling. This argument is further corroborated by the same overall trend being reproduced on a second sample, fabricated in the same way and cycled with a close to identical hydrogenation scheme (see **Supplementary Figure 20b** and Supplementary Data).

It is interesting to note that the inflection point of this cyclic behavior, i.e. the cycle where the average $P_{abs}$ switches from monotonically increasing to monotonically decreasing, seems to be sample specific but also compression-level independent. On the primary sample (**Supplementary Figure 14a**), the inflection point for *both* compressed and non-compressed particles is around cycle 50, while for the second sample (**Supplementary Figure 20b**) the inflection point is already at cycle 40. A partial answer to this initially non-intuitive trend can be glanced from phase IV-V of the $P_{abs}$ evolution (**Supplementary Figure 14a**). In this final part of the hydrogenation procedure, three absorption isotherms were measured one after another in consecutive cycles (see **Supplementary Figure 14e-f**) for the individual isotherms for two non-compressed particles). During these measurements, the average $P_{abs}$ again go through the same cyclic behavior, where $P_{abs}$ initially increases, peaks and then decreases again. This cyclic behavior is much more obvious in phase IV-V when $P_{abs}$ is plotted against the total time spent in the hydrogenated state instead of number of hydrogenation cycles (**Figure 3c** of the main text and **Supplementary Figure 14b**). As such, this implies that it is the total time spent in the hydrogenated state that dictate the frequency of the cyclic $P_{abs}$ behavior, potentially mediated by a cyclic dislocation formation and annihilation that is closely related to the stochastic nature of dislocation

substructure in the nanoparticles, which in turn is strongly dependent on their fabrication pre-history and compression degree. Why the formation of new dislocations would not enhance the kinetics might at first seem counterintuitive, but here it is important to remind ourselves about the different energy barriers involved. For the thermodynamic absorption plateau pressure, the strain of the overall system during hydrogenation decides $P_{abs}$ (also potentially influenced by specific dislocations that can act as nucleation sites for the hydride phase)[13-15], while the kinetically rate-limiting step of the hydrogen absorption process in Pd is the diffusion of dissociated hydrogen from the surface to the first sub-surface layer[16,17]. This means that hypothetically, dislocations could form and annihilate in a region of the particle far from the surface, *e.g.* close to the highly strained substrate-particle interface, and thus modify $P_{abs}$ in the process, while dislocations close to the free surface continuously annihilate during the hydrogenation procedure and kinetics accordingly continuously decrease.

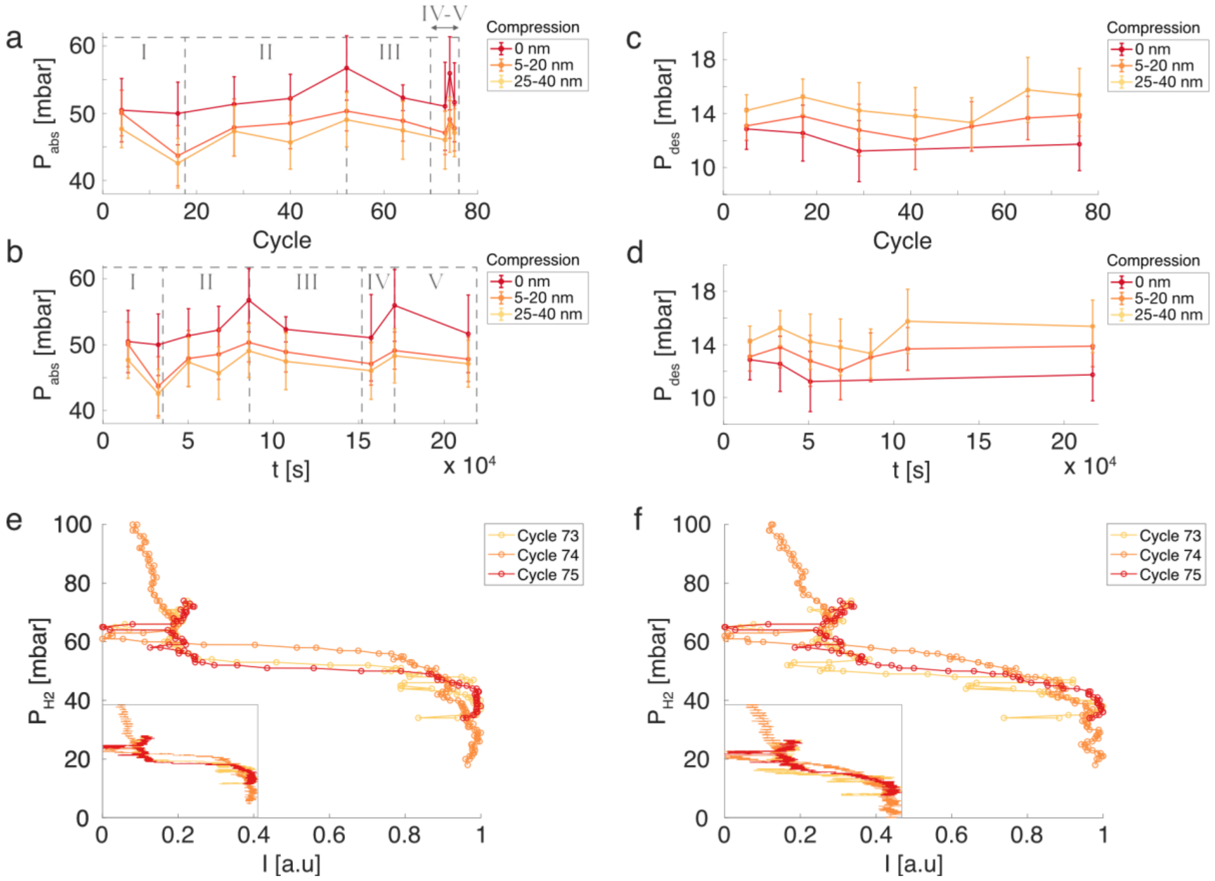


**Supplementary Figure 14.** *The evolution of the average absorption, $P_{abs}$, (a-b) and desorption, $P_{des}$ (c-d) plateau pressures with the particles divided into three groups depending on their degree of compression, i.e., non-compressed (0 nm), 5-20 nm compression and 25-40 nm compression plotted as a function of hydrogenation cycle (a,c) and as a function of time spent in > 40 mbar $H_2$ (b,d). The error bars indicate one standard deviation calculated from the individual plateau pressures of the particles within each compression group. To have comparable results between the compressed (5-20 nm and 25-40 nm group) and the non-compressed (0 nm) groups of particles, only particles in the non-compressed group (0 nm) with $t_{30\text{-}50}$ hydrogen absorption times comparable to the particles in the compressed groups have been used in (a,b). For more information, see SI section 11. The intermediate cycles (75 > cycle > 30) for the non-compressed particles in (c,d) were left out in accordance with the discussion in SI section 11. The evolution of the absorption plateau pressures is divided into five different phases (I-V) which are explained in more details in the main text of this section. (e-f) Average dark-field scattering intensity profiles from two individual non-compressed Pd particles (e and f respectively) during 3 consecutive absorption isotherm measurements. The average intensity profile is calculated such that we get two (averaged) data points per hydrogen concentration, e.g. if every hydrogen concentration is kept for 300 s, the first (averaged) intensity data point is the average from 1-150 s and the second is the*

*average from 151-300s. The two chosen particles have $t_{30\text{-}50}$ hydrogen absorption times comparable to the particles in the compressed groups. Inset: Raw dark-field scattering intensity profiles.*

## 13. Kinetics deacceleration model

**Supplementary Figure 15** presents the fit of an exponential model $t_{30-50} = t_0 e^{kx}$, where $x$ is the hydrogenation cycle, to the $t_{30\text{-}50}$ absorption time data for the non-compressed (0 nm) particles, the 5-10 nm compressed particles (grouped together for additional data points), and the 35-40 nm compressed particles (also grouped together). To make sure any oxides on the surface of the particles are sufficiently reduced (see SI section 6 regarding reduction of oxides), the first cycle included in the fit is cycle 10. From this fit, we note that (i) the particles exhibit an initial absorption time at cycle 10 ($t_0$, **Supplementary Figure 15c**) that is proportional to their compression degree and (ii) the non-compressed particles have a significantly higher de-acceleration rate $k$ compared to the two groups of compressed particles ($k$, **Supplementary Figure 15d**). We attribute this difference to a transition for the compressed particles from a dislocation starvation regime to more of a forest hardening regime (see SI section 3). In other words, the compression introduces so many new dislocations in the particles that dislocation pinning might come into effect, i.e. it becomes "harder" for the dislocations to heal out compared to dislocations in the non-compressed particles, which in turn leads to the different deaccleration rates between compressed and non-compressed particles.

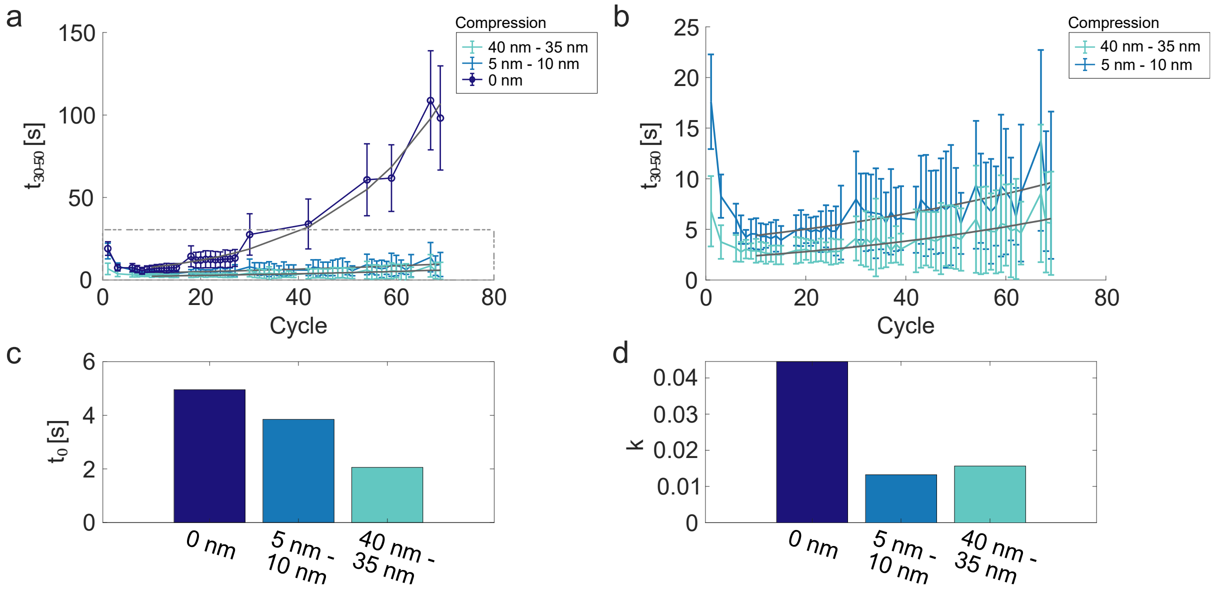


**Supplementary Figure 15.** *Exponential deacceleration model fit to the experimental data. (a) The model $t_{30-50} = t_0 e^{kx}$ fitted to the non-compressed (0 nm) particles, the 5-10 nm compressed particles (grouped together for additional data points), and the 35-40 nm compressed particles (also grouped together). The error bars indicate one standard deviation calculated from the individual $t_{30\text{-}50}$ absorption times of the particles within each compression-level sub-group. (b) Magnified view of the fit on the compressed particle sub-groups in panel a (dashed box). (c-d) The fitted model parameters, $t_0$ (c) and $k$ (d) for the three different sub-groups.*

## 14. Statistical analysis of hydrogenation kinetics and isothermal plateau pressures

To check whether or not the effect of nanocompression to different degrees have a statistically significant effect on the hydrogenation kinetics of the particles, a Student's t-test on the $t_{30\text{-}50}$ kinetics absorption times on the two extremes of the compressed particles, i.e. the 5-10 nm compressed particles (grouped together for additional data points), and the 35-40 nm compressed particles (also grouped together for additional data points) was performed. The null hypothesis for this statistical t-test is that the data points are independent random samples from normal distributions with equal means and equal but unknown variances. The alternative hypothesis is that the data comes from populations with unequal means. **Supplementary Figure 16** shows for which cycles the test rejects the null hypothesis at the 5% significance level (highlighted with filled squares at the corresponding data points). The average *P*-value for the cycles that passed the test (i.e. *P*-values < 0.05) was 0.0084 and the average degrees of freedom for all tested cycles was 27.38 (standard deviation 1.45). This test shows that the difference in absorption kinetics are indeed significant between the two extremes of the compressed particles (i.e. the 5-10 nm and the 35-40 nm compressed particles), at least for the first ~50 cycles.

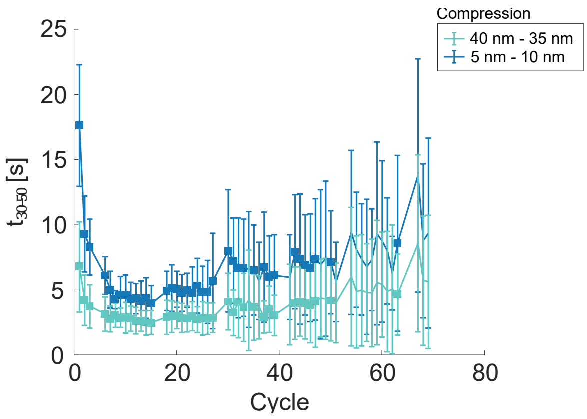


**Supplementary Figure 16.** *Student's t-test hypothesis testing on the two extremes of the compressed particles, i.e. the 5-10 nm compressed particles, and the 35-40 nm compressed particles. For cycles where the null hypothesis, i.e. that the kinetics of the 5-10 nm and the 35-40 nm compressed particles are represented with the same distribution with equal means, is rejected at the 95% confidence level is highlighted with filled squares. The error bars indicate one standard deviation calculated from the individual $t_{30\text{-}50}$ absorption times of the particles within each compression-level sub-group.*

A similar Student's t-test was also performed on the absorption plateau pressure data. Here we compared all three compression groups of **Figure 3b-c** of the main text with each other (i.e. comparing the 0 nm compressed particles to the 5-20 nm compressed particles, comparing the 0 nm compressed particles to the 25-40 nm compressed particles and finally also comparing the 5-20 nm compressed particles to the 25-40 nm compressed particles). **Supplementary Figure 17** shows for which cycles the test rejects the null hypothesis at the 5% significance level (highlighted with filled squares at the corresponding data points). The average *P*-value for the cycles that passed the test (i.e. *P*-values < 0.05) was 0.0093 (**a**), 0.0037 (**b**) and the degrees of freedom for all tested cycles were [312, 296,149, 110, 37, 25, 30, 30, 30] (**a**), [318, 302, 155, 116, 43, 31, 36, 36, 36] (**b**) and 54 (**c**). The reason for the drastic decrease in degrees of freedom for the later cycles in (**a**,**b**) is due to the influence of the slowing kinetics on the isotherm measurements described in section 11. From these tests it's clear that there is a statistical significant difference between the absorption plateau pressures for the non-compressed particles and both groups of compressed particles. However, that there would be a statistical significant difference between the absorption plateau pressures between the two groups of compressed particles is less clear.

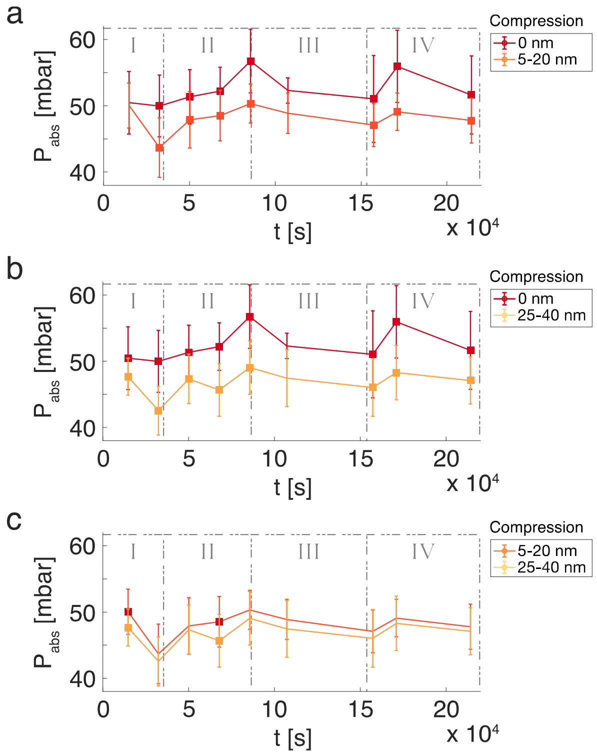


**Supplementary Figure 17.** *Student's t-test hypothesis testing on the absorption plateau pressures for three groups of differently compressed particles; (a) the 0 nm compressed particles compared to the 5-20 nm compressed particles, (b) the 0 nm compressed particles compared to the 25-40 nm compressed particles and (c) the 5-20 nm compressed particles compared to the 25-40 nm compressed particles. For cycles where the null hypothesis, i.e. that the absorption plateau pressures are represented with the same distribution with equal means, is rejected at the 95% confidence level is highlighted with filled squares. The error bars indicate one standard deviation calculated from the individual plateau pressures of the particles within each compression group.*

## 15. SEM imaging and kinetic data of selected particles from the main text

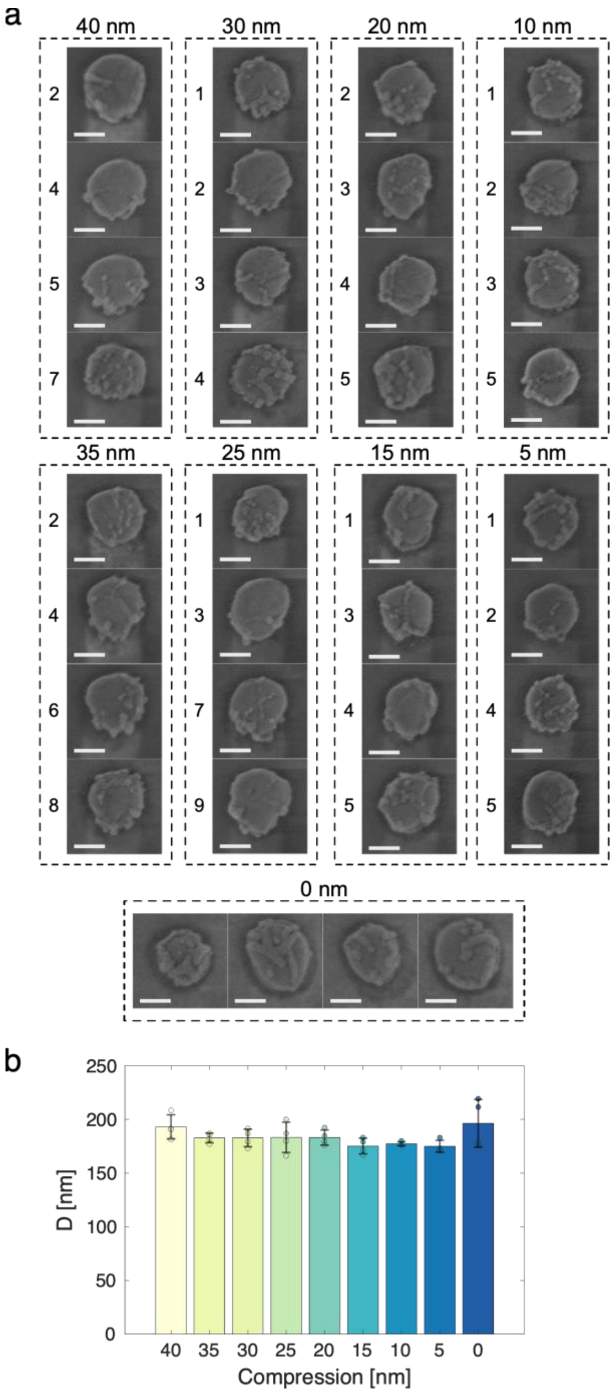


**Supplementary Figure 18.** *(a) SEM micrographs of 4 randomly selected particles from each compression-level subgroup after all 76 cycles of hydrogen cycling. The numbers correspond to the particle numbering of* ***Supplementary Figure 19****. Scale bars are 100 nm. (b) Average diameter (calculated from the mean of the horizontal and vertical diameter) of the imaged particles in (a) as a function of compression level. Error bars represent one standard deviation. The individual data points are also plotted as filled circles.*

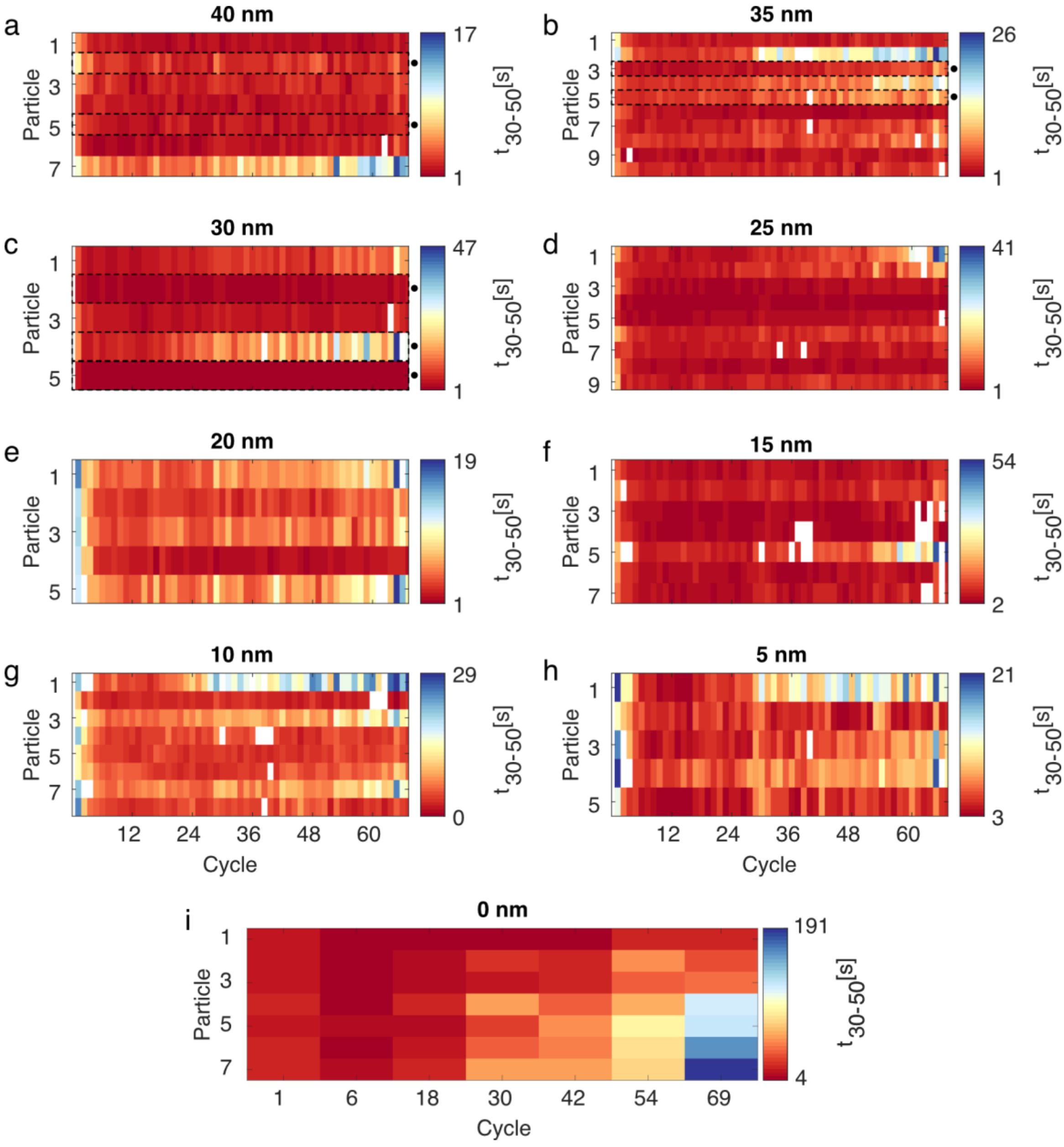


**Supplementary Figure 19.** *The individual $t_{30\text{-}50}$ values measured for every compressed particle for all hydrogenation cycles and for 7 selected non-deformed particles (0 nm) for selected cycles. The deformation degree is indicated on top of every panel and the $t_{30\text{-}50}$ time for each cycle and nanoparticle is represented by a color-code shown in the color bar to the right of each plot. If the color for a certain particle and cycle is white, then the signal for this particle was discarded during the data analyzation step due to the particle not having desorbed completely from the last cycle before the next hydrogenation (for more information, see section 10). Particles that were nominally single-crystalline (i.e. showed a strain-burst in their load-displacement diagrams) are highlighted with a black dot, as well as a dashed box. The 7 non-deformed particles (0 nm) were chosen to have final absorption times representative of the wide distribution seen for the non-compressed particles in* ***Figure 5c*** *of the main text.*

## 16. Kinetic and isotherm data from SEM-imaged particles on second sample

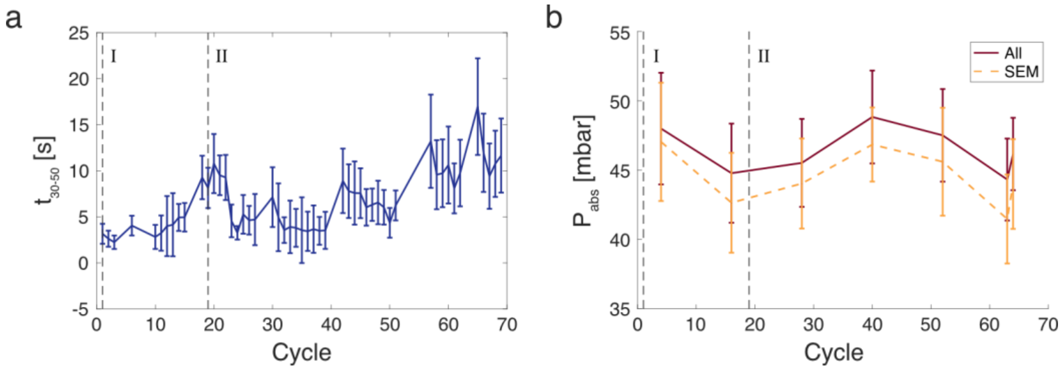


**Supplementary Figure 20.** *The evolution of the average $t_{30-50}$ absorption times (a) and average absorption $P_{abs}$ plateau pressures (b) for the 44 SEM imaged particles in* ***Supplementary Figure 1a*** *as a function of hydrogenation cycles. In b, the average absorption ($P_{abs}$) plateau pressures for the rest of the particles on the sample are also plotted. The sample (S2) was fabricated in the same way as the sample discussed in the main text (S1) - except that no particles have been compressed. The particles have been cycled using similar hydrogenation procedure as for the sample discussed in the main text (S1), see Supplementary Data 1. The time of the two SEM imaging sessions that give rise to the two sets of images in* ***Supplementary Figure 1a*** *are indicated in both plots with dashed lines (I and II respectively). We note that the emergence of the protrusions between set I and set II discussed in* ***Supplementary Figure 1a*** *correspond with a deacceleration of kinetics (a) and a decrease in absorption ($P_{abs}$) plateau pressure (b). As in the main text, we attribute these changes to the annihilation of existing dislocations (deacceleration of kinetics) together with a reduction of stress levels (decrease in absorption plateau pressure $P_{abs}$)*[15] *and are as such consistent with the discussion in the main text, where the growth of protrusions on the particles are described as a stress-relief mechanism. The error bars indicate one standard deviation calculated from the individual $t_{30-50}$ absorption times (a) and plateau pressures (b) of the particles within each compression group.*

## 17. Kinetic and isotherm data of STEM analyzed particles of the main text

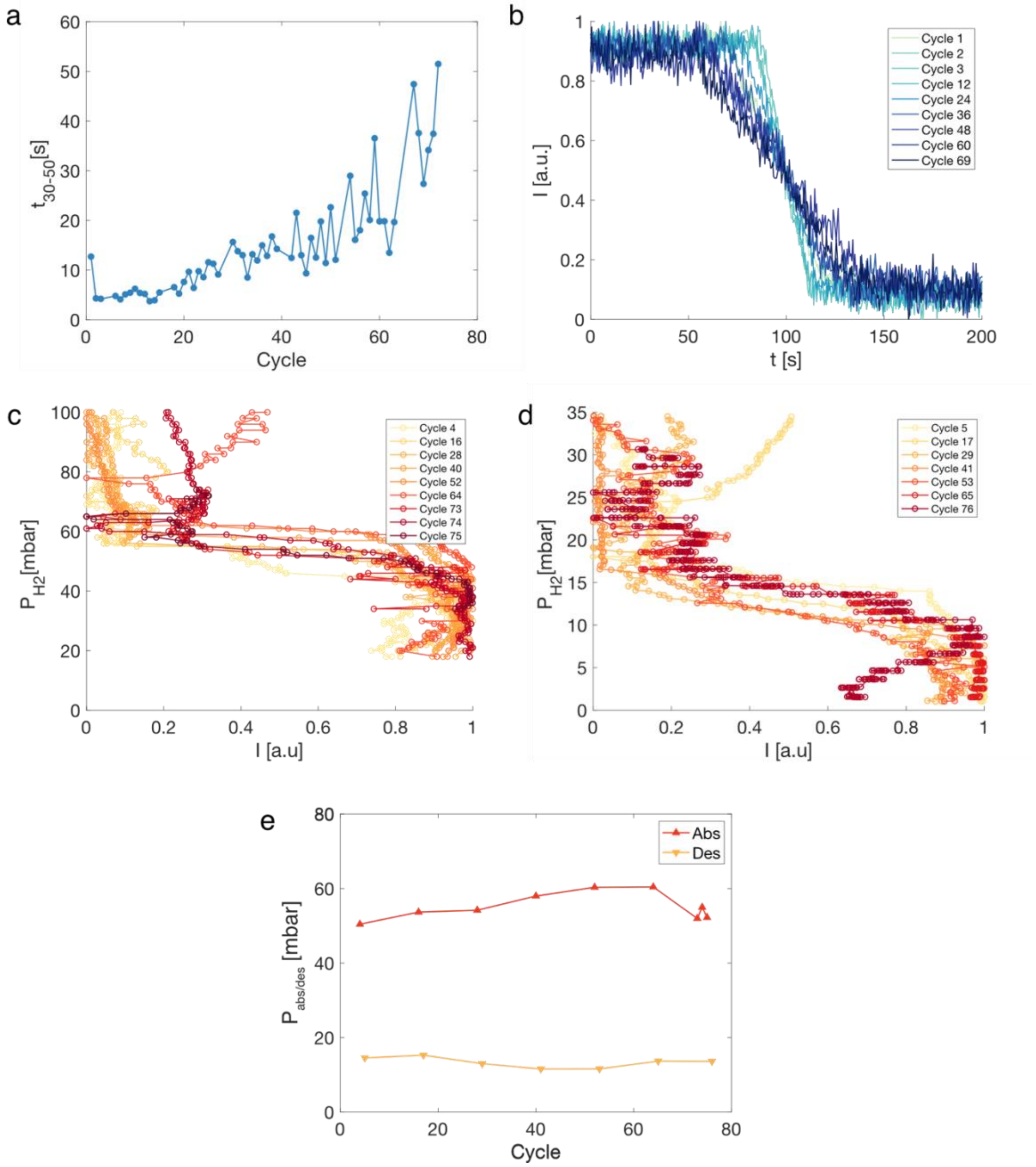


**Supplementary Figure 21.** *Hydrogenation properties of the particle in* ***Figure 4d-f*** *in the main text. (a) Absorption time $t_{30-50}$ evolution as a function of hydrogenation cycles. (b) Dark-field scattering intensity profiles from which the absorption times in (a) were calculated for a select number of hydrogenation cycles. (c-d) Hydrogen partial-pressure vs average dark-field scattering intensity isotherms for absorption (c) and desorption (d). The average intensity profile is calculated such that we get two (averaged) data points per hydrogen concentration, e.g. if every hydrogen concentration is kept for 300 s, the first (averaged) intensity data point is the average from 1-150 s and the second is the average from 151-300s. (e) Absorption ($P_{abs}$) and desorption ($P_{des}$) plateau pressures as a function of hydrogenation cycles calculated from the intensity-pressure isotherms in c-d.*

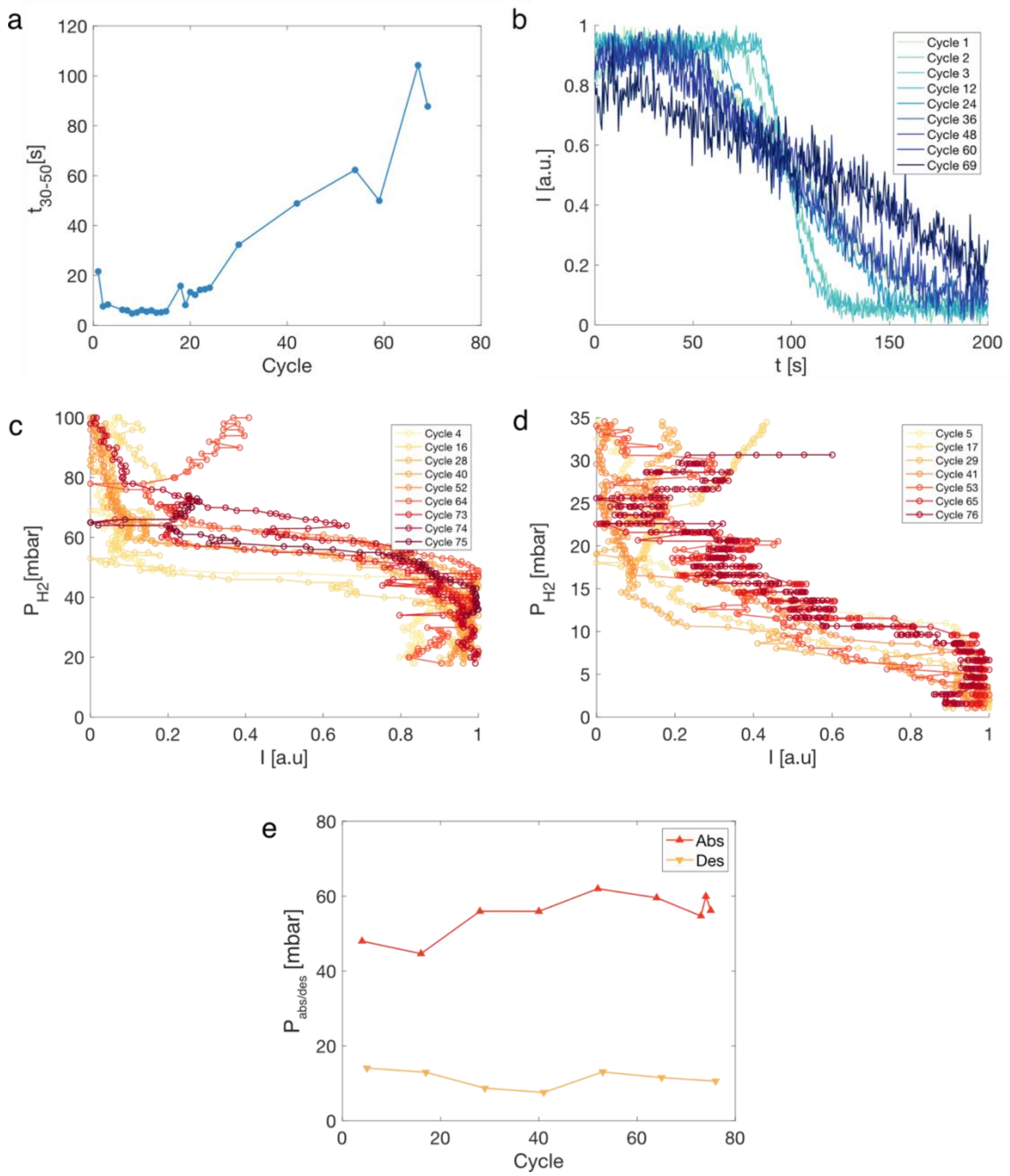


**Supplementary Figure 22.** *Hydrogenation properties of the particle in* ***Figure 4g-i*** *in the main text. (a) Absorption time $t_{30\text{-}50}$ evolution as a function of hydrogenation cycles. (b) Dark-field scattering intensity profiles from which the absorption times in (a) were calculated for a select number of hydrogenation cycles. (c-d) Hydrogen partial-pressure vs average dark-field scattering intensity isotherms for absorption (c) and desorption (d). The average intensity profile is calculated such that we get two (averaged) data points per hydrogen concentration, e.g. if every hydrogen concentration is kept for 300 s, the first (averaged) intensity data point is the average from 1-150 s and the second is the average from 151-300s. (e) Absorption ($P_{abs}$) and desorption ($P_{des}$) plateau pressures as a function of hydrogenation cycles calculated from the intensity-pressure isotherms in c-d.*

## 18. Estimation of stress during hydrogenation

Before we estimate the stress experienced by the particles during hydrogenation, we must emphasize that this stress will be individual in each particle, as the consequence of several simultaneously acting parameters, i.e., substrate clamping, individual particle morphology and hydrogenation history. Hence, it is not realistic to attempt any description that captures the behavior of each individual particle (and deformation level). However, we can provide a very rough estimate for an averaged response of all particles. We make use of the Young's elastic modulus of Pd (121 GPa) together with as-of-yet unpublished results from our group (the study is in manuscript form), including in situ AFM measurements of the volume expansion during hydrogenation of Pd nanoparticles very similar to the ones studied here and nanofabricated in the same way.[18] In this AFM study, the hydrogenation resulted in a ~ 11% volume expansion (which is comparable to the 10 % volume expansion reported for bulk Pd[19]). Assuming fully homogenous stress levels during hydrogenation, which technically only holds for unsupported, defect-free, single-crystalline particles, and that the stress-strain relationship follows Hooke's law together with an isotropic Young's elastic modulus – then yields an approximate linear hydrogenation stress of 4.4 GPa. We want to emphasize, that beyond a large variation in the particle-to-particle hydrogenation stress levels, which is a consequence of individual particle morphologies and microstructures, we also have additional conditions that could lead to this approximate hydrogenation stress being both under- or overestimated respectively, e.g. plastic relaxation processes during the hydrogenation or increased stress levels due to strong substrate clamping by the sapphire substrate. Consequently, we conservatively only state that the stress levels during hydrogenation are in the GPa range. Notably, this stress level is indeed comparable to the stress levels reached during the hydrogenation of annealed and nano-crystalline Pd thin films by Delmelle R. et al.[20], where this level of stress was sufficient to activate dislocation-nucleated plasticity in both the annealed and non-annealed samples.

## 19. Full width at half maximum for every individual compression sub-group

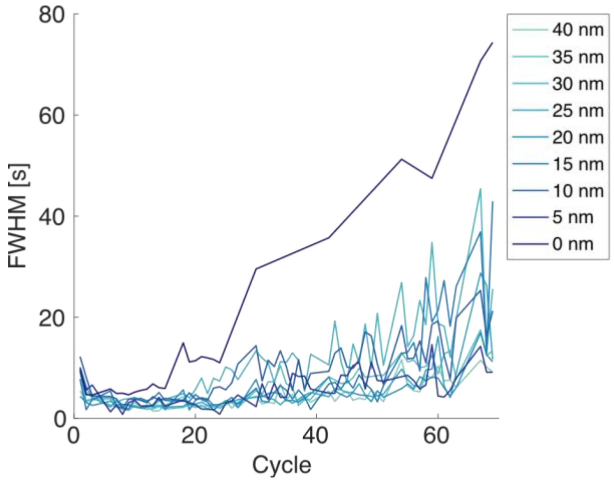


**Supplementary Figure 23.** *The full width half maximum (FWHM) of the $t_{30\text{-}50}$ absorption time distributions for all compression-level sub-groups as calculated from a normal distribution fitted to the $t_{30\text{-}50}$ histograms of each individual sub-group.*

## 20. Histograms of the single particle absorption plateau pressures $P_{abs}$

By fitting a normal distribution to the histograms of the single particle absorption plateau pressures $P_{abs}$ for individual cycles (**Supplementary Figure 24a**), we note what seems to be a significant discrepancy

in how the full width at half maximum (FWHM) evolves for the distributions for the compressed (5-20 nm as well as the 25-40 nm compressed group) vs the non-compressed (0 nm compression) particles. For the non-compressed particles, the FWHM decreases with cycle number, i.e. the particles converge towards an average absorption plateau pressure $P_{abs}$, which can be interpreted as the non-compressed particles converging toward a thermodynamic equilibrium for the absorption plateau pressure. This convergence towards an average $P_{abs}$ is also very clear directly from the histograms for the non-compressed particles (**Supplementary Figure 24b**) and is also reproduced on a second sample (**Supplementary Figure 30**).

For the compressed particles on the other hand, we instead see an initial increase of the distribution FWHM, before eventually decreasing (**Supplementary Figure 24a**). The inflection point, i.e. the cycle where the FWHM stops increasing and starts to decrease, also seems to be compression-level dependent, i.e. the FWHM of the most compressed particles (25-40 nm compressed group) peaks at a later cycle than the least compressed group of particles (5-20 nm compressed group). We tentatively attribute these distinct compression-level trends to the plastic deformation induced by the microcompression, where the individual dislocation networks created in every compressed particle initially decide the $P_{abs}$ evolution of these particles during hydrogen cycling – mediated either through the dislocations directly acting as nucleation sources for the β-phase or the individual strain levels of the particles – leading to more diverse $P_{abs}$ values. In other words, the more compressed the particle, the more time it needs to spend in the high-diffusivity hydrogenated state before reaching the thermodynamic equilibrium. Finally, we should also add that the increase in FWHM for the compressed sub-groups (5-20 nm and 25-40 nm) may also only be apparent since the changes in width of the distributions for different cycles are very small and the amount of data is limited (see **Supplementary Figure 24c-d**).

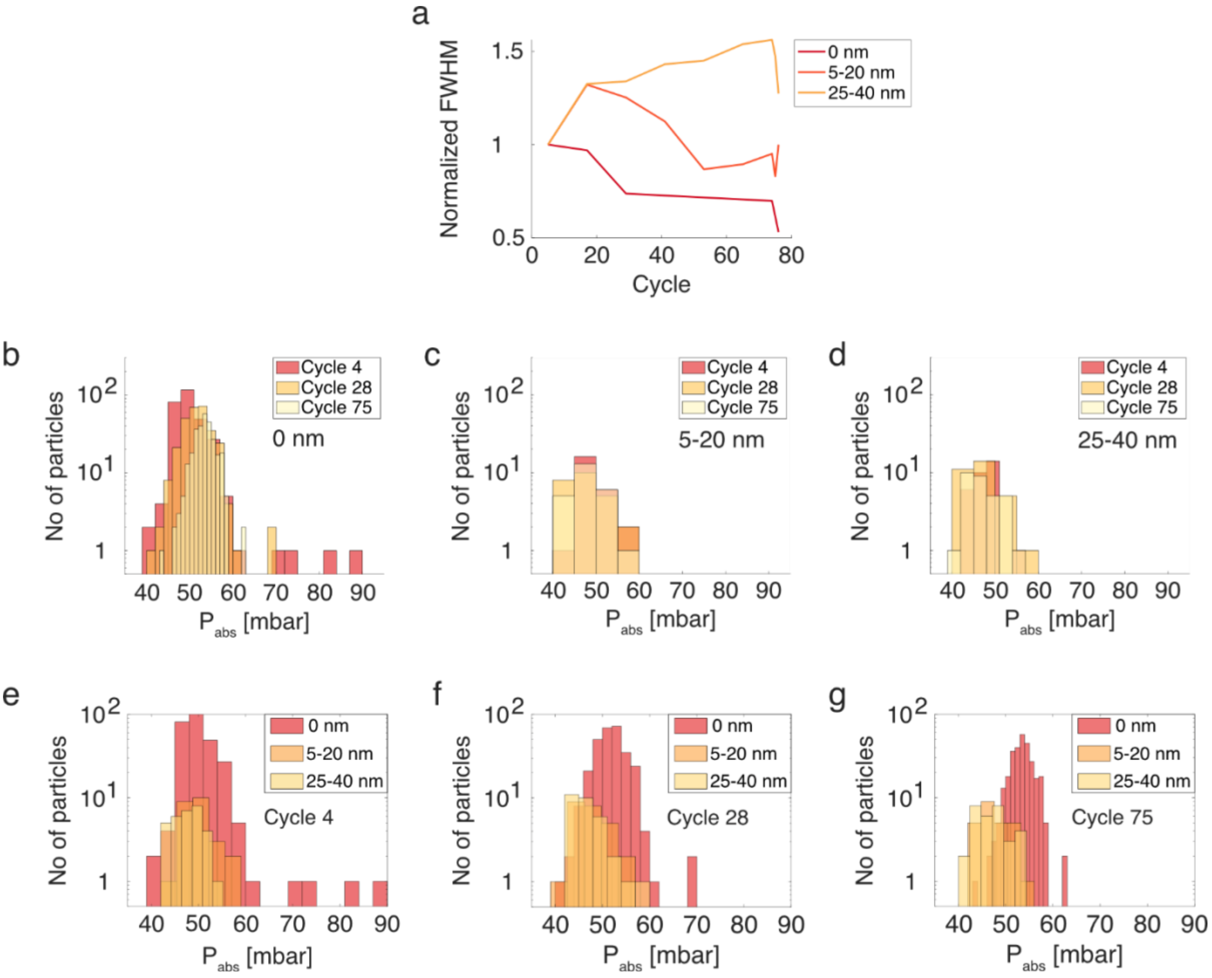

**Supplementary Figure 24.** *(a) The full width half maximums (FWHM) of the hydrogen absorption plateau pressure distributions (as calculated from a normal distribution fitted to the* $P_{abs}$ *histograms) normalized to the FWHM of the corresponding first* $P_{abs}$ *measurement with particles divided into three groups depending on their degree of compression, i.e., non-compressed (0 nm), 5-20 nm compression and 25-40 nm compression. (b-d) Histograms of the single particle absorption plateau pressures* $P_{abs}$ *for three specific cycles. The particles are divided into three groups depending on their degree of compression, i.e., non-compressed (0 nm) (b), 5-20 nm compression (c) and 25-40 nm compression (d). We note the distinct decrease in distribution width for the non-compressed sub-group for later hydrogenation cycles (b). For the compressed sub-groups (c,d), any change to the distribution width is not as apparent. (e-g) Histograms of the single particle absorption plateau pressures* $P_{abs}$ *for cycle 4 (e), 28 (f) and 75 (g). The particles are divided into three groups depending on their degree of compression, i.e., non-compressed (0 nm), 5-20 nm compression and 25-40 nm compression.*

## 21. Comparison of kinetics between initially single and polycrystalline particles

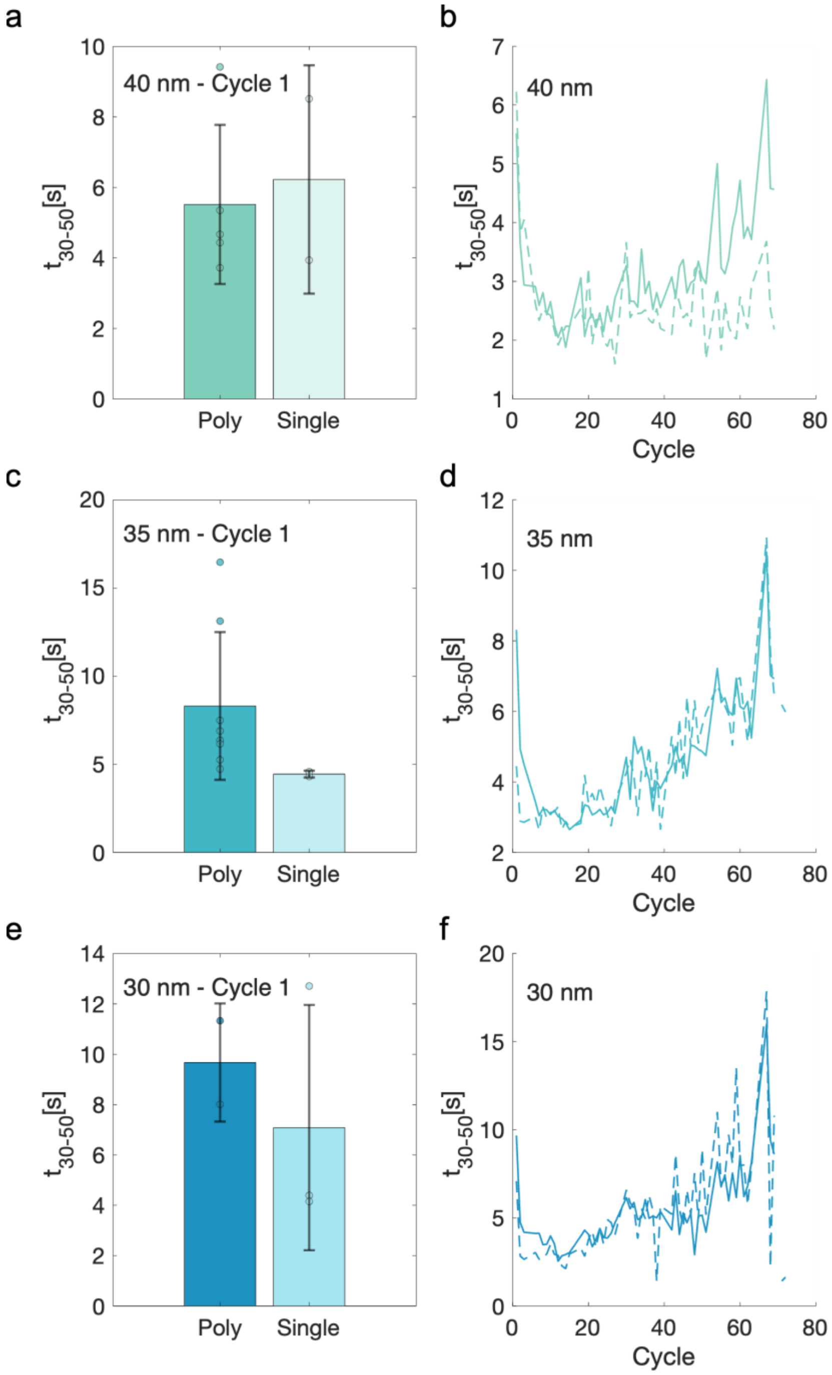


**Supplementary Figure 25.** *Comparison of $t_{30\text{-}50}$ values for single (strain burst) vs. polycrystalline (staircase-yielding) particles. See **Supplementary Figure 2** for the individual designations for every particle. The particles are from the 40 nm (a-b), 35 nm (c-d) and 30 nm (e-f) compressed sub-groups. The left column (a,c,e) shows the mean $t_{30\text{-}50}$ values for nominally single vs polycrystalline particles for hydrogenation cycle 1. The error bars indicate one standard deviation calculated from the individual $t_{30\text{-}50}$ values for the particles within each group. The individual data points are also plotted as filled circles. The right column (b,d,f) shows the evolution of the mean $t_{30\text{-}50}$ values for nominally single crystalline (dashed lines) and nominally polycrystalline (solid lines) particles as a function of hydrogenation cycles.*

# 22. Single particle comparison between kinetics and absorption plateau pressures

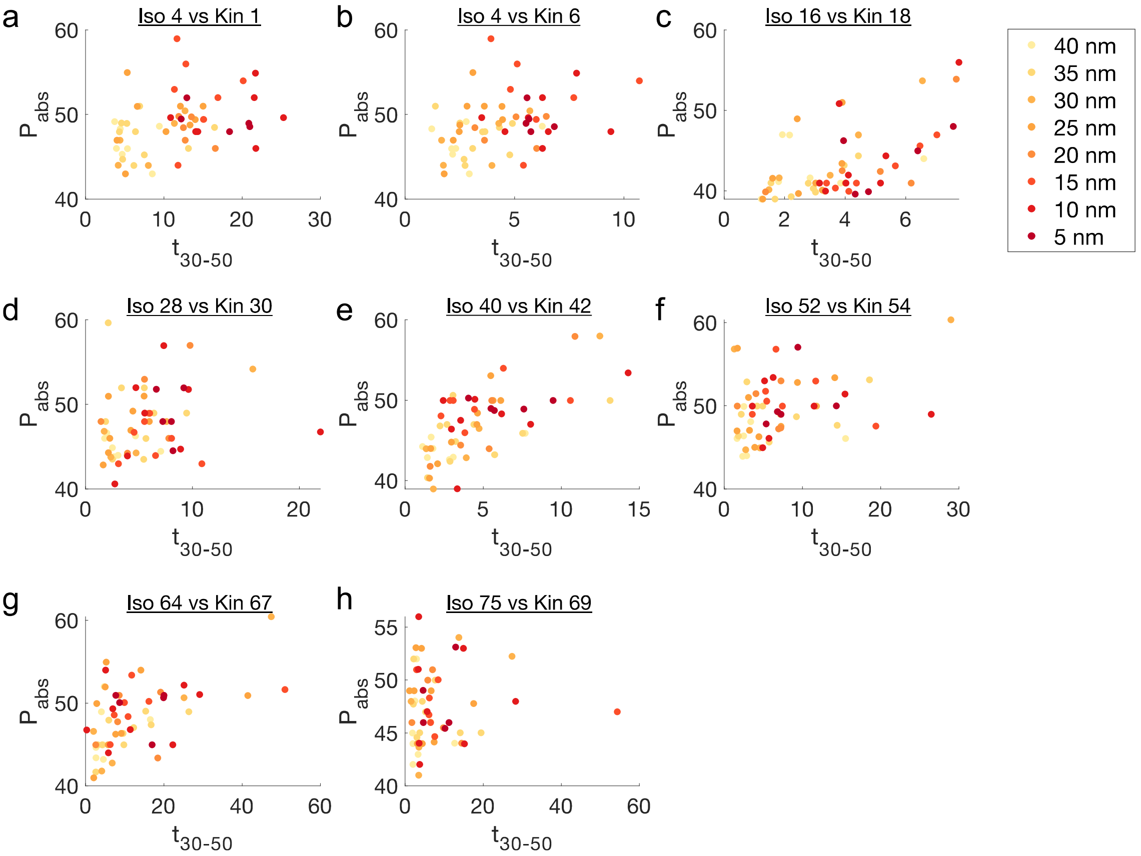


**Supplementary Figure 26.** *Absorption plateau pressures $P_{abs}$ (mbar) vs. $t_{30\text{-}50}$ (s) absorption times for all compressed particles. Stated above each plot is the hydrogenation cycle number for the isotherm (Iso) and the kinetics measurement (Kin), respectively. Particles are color-coded according to their individual degree of compression.*

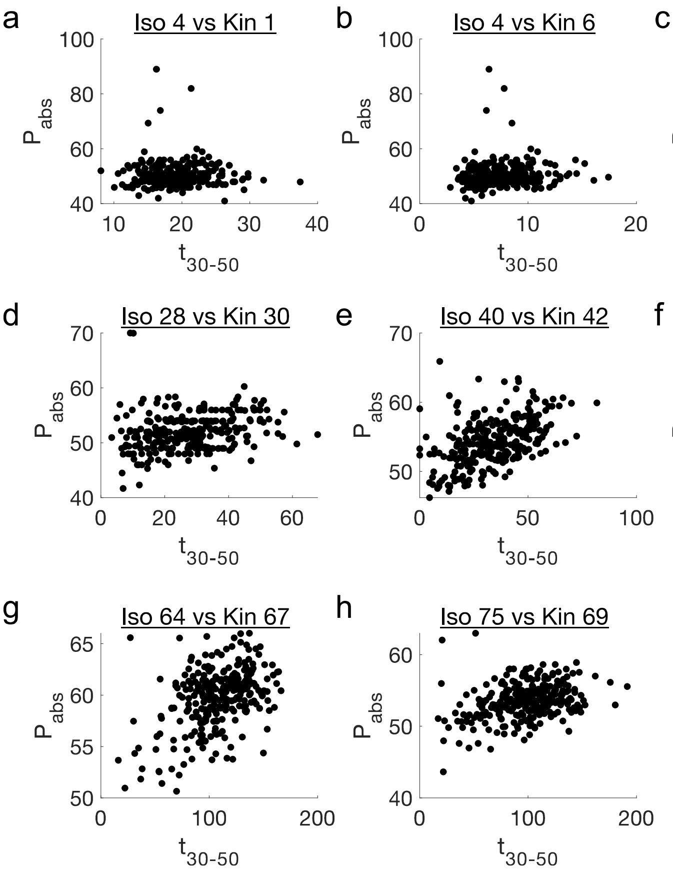


**Supplementary Figure 27.** *Absorption plateau pressures $P_{abs}$ (mbar) vs $t_{30\text{-}50}$ (s) absorption times for all non-compressed particles. Stated above each plot is the hydrogenation cycle number for the isotherm (Iso) and the kinetics measurement (Kin), respectively.*

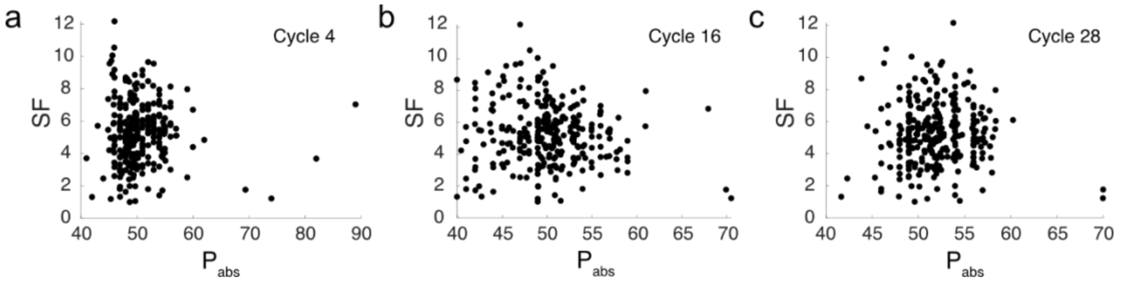


**Supplementary Figure 28.** *Slowing factor SF (defined as the ratio between the $t_{30\text{-}50}$ absorption times for cycle 69 and cycle 1) of all non-compressed particles vs their absorption plateau pressures $P_{abs}$ (mbar) for cycle 4 (a), 16 (b) and 28 (c).*

# 23. Comparison of results between main text and second sample

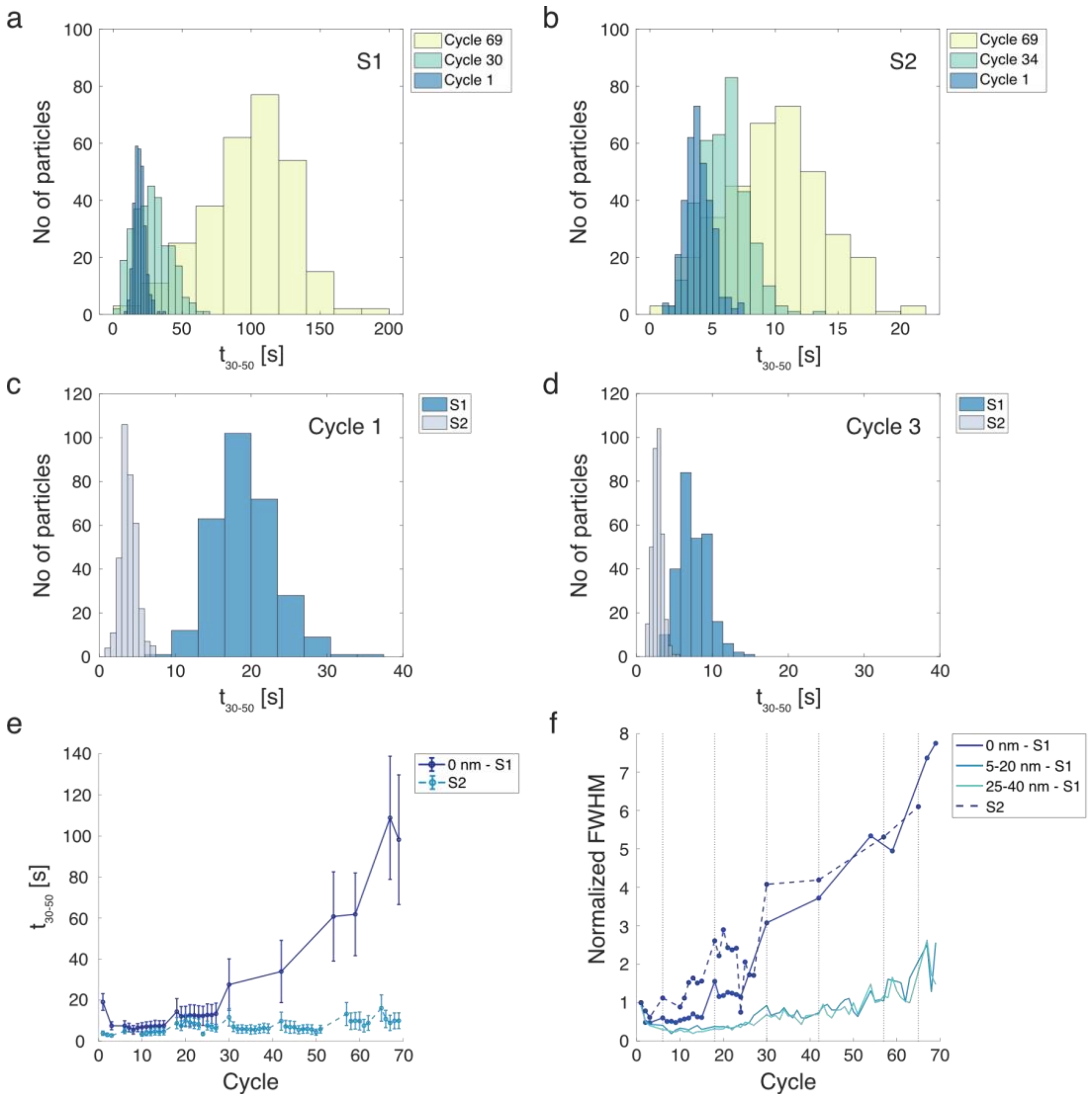


**Supplementary Figure 29.** *Comparison of the hydrogen absorption kinetics for sample S1 of the main text and sample S2. S2 was fabricated in the same way as the sample discussed in the main text (S1) - except that no particles have been compressed. The particles have been cycled using similar hydrogenation procedure as S1, see Supplementary Data 1. (a-b) Histograms of the single particle $t_{30\text{-}50}$ absorption times for S1 (a) and S2 (b) for three specific cycles. We note how the $t_{30\text{-}50}$ distributions for both samples widen and shift to slower absorption times at later cycles. (c-d) Comparisons of the single particle $t_{30\text{-}50}$ histograms for S1 and S2 for cycle 1 (c) and cycle 3 (d) respectively. (e) The evolution of the average $t_{30\text{-}50}$ absorption times for the non-compressed particles (0 nm) of sample S1 and the particles of sample S2 (all of which are non-compressed). The error bars indicate one standard deviation calculated from the individual $t_{30\text{-}50}$ absorption times of every particle within each compression group and sample. (f) The full width half maximums (FWHM) of the $t_{30\text{-}50}$ absorption time distributions for S1 and S2 (as calculated from a normal distribution fitted to the $t_{30\text{-}50}$ histograms) normalized to the FWHM of the corresponding first cycles. The particles of S1 are divided into three groups depending on their degree of compression, i.e., non-compressed (0 nm), 5-20 nm compression and 25-40 nm compression. The method explained in section 10 for only taking data into account where most particles have fully desorbed resulted in mostly cycles right after isotherms making the cut for the non-compressed (0 nm) particles on S1. Therefore, the corresponding cycles are also plotted for S2 to*

*ensure comparability. We note that both sets of non-compressed particles (0 nm S1 and S2) develop comparatively broader distributions than their compressed counterparts.*

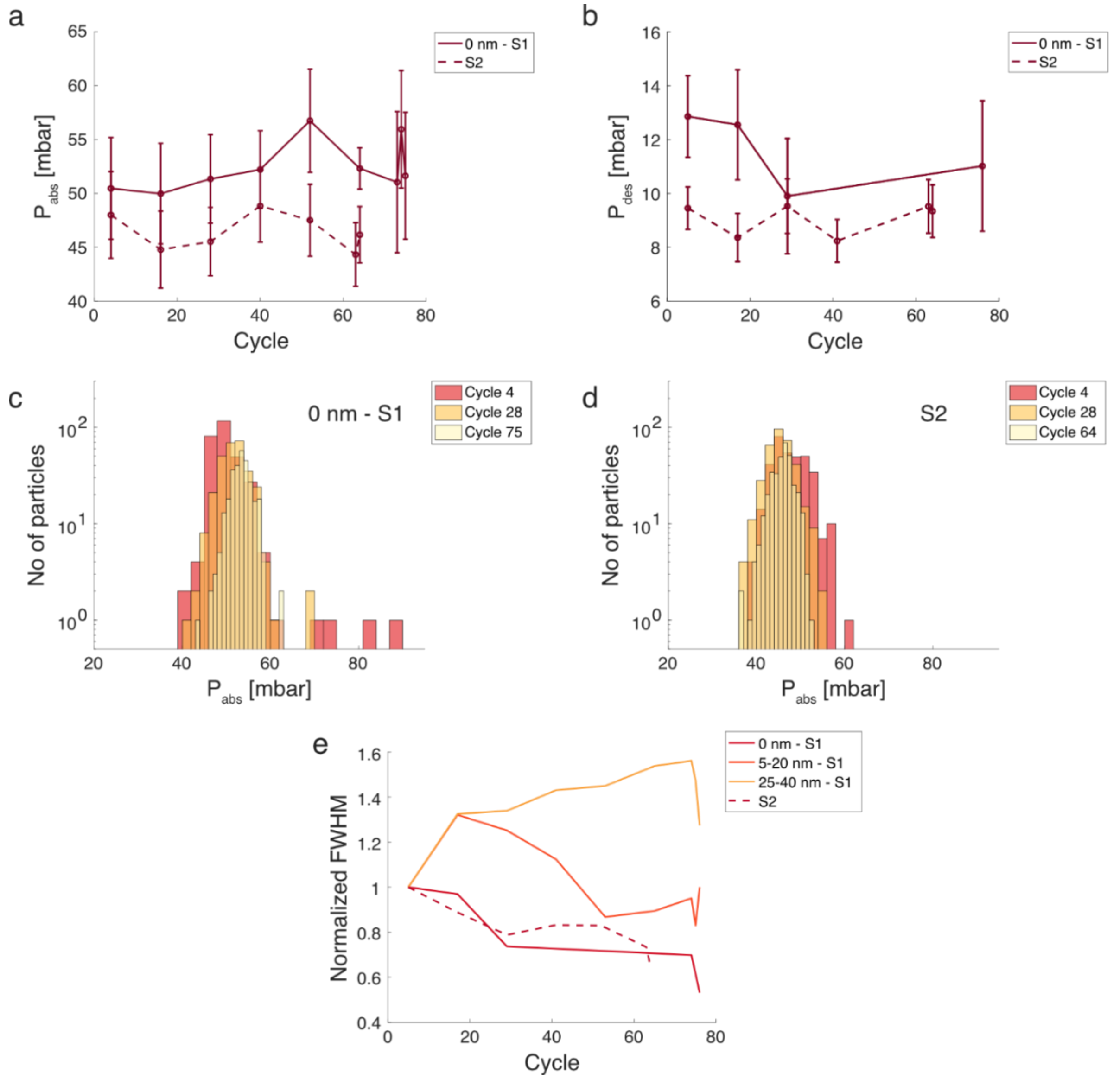


**Supplementary Figure 30.** *(a-b) Comparison of the average hydrogen absorption (a) and desorption (b) plateau pressures for the non-compressed (0 nm) particles of sample S1 of the main text and sample S2. S2 was fabricated in the same way as the sample discussed in the main text (S1) - except that no particles have been compressed. The particles have been cycled using similar hydrogenation procedure as S1, see Supplementary Data 1. For S1 in a, only particles with absorption times comparable to the compressed particles ($t_{30\text{-}50}$< 25 s) were included and for b, the intermediate cycles (75 > cycle > 30) were left out in accordance with the discussion in section 11. The error bars indicate one standard deviation calculated from the individual plateau pressures of the particles within each compression group and sample. (c-d) Histograms of the single particle $P_{abs}$ absorption times for the non-compressed (0 nm) particles of sample S1 (c) and S2 (d) for three specific cycles. We note how the width of the distributions for both samples decrease at later cycles. (e) The full width half maximums (FWHM) of the hydrogen absorption plateau pressure distributions for S1 and S2 (as calculated from a normal distribution fitted to the $P_{abs}$ histograms) normalized to the FWHM of the corresponding first $P_{abs}$ measurement. We note that for both non-compressed populations (S2 and S1 – 0 nm), the relative distribution width decrease for later cycles, i.e. the $P_{abs}$ values (and thereby the particle strain levels according to the SKG-model) tend to converge towards a single $P_{abs}$ value (that is distinct for each*

*population). The compressed sub-groups on the other hand (5-20 nm S1 and 25-40 nm S1), instead show a trend where the width of the distributions initially increase before decreasing at later cycles, with the less compressed sub-group (5-20 nm S1) peaking earlier and at a lower value than the more compressed sub-group (25-40 nm).*

**Supplementary References**


1 El-Awady, J. A. Unravelling the physics of size-dependent dislocation-mediated plasticity. *Nature communications* **6**, 5926 (2015). https://doi.org/10.1038/ncomms6926

2 Zimmerman, J. *et al.* Drastic softening of Pd nanoparticles induced by hydrogen cycling. *Scripta Materialia* **253** (2024). https://doi.org/10.1016/j.scriptamat.2024.116304

3 Mordehai, D. *et al.* Size effect in compression of single-crystal gold microparticles. *Acta Materialia* **59**, 5202-5215 (2011). https://doi.org/10.1016/j.actamat.2011.04.057

4 Sharma, A., Hickman, J., Gazit, N., Rabkin, E. & Mishin, Y. Nickel nanoparticles set a new record of strength. *Nature Communications* **9**, 4102 (2018). https://doi.org/10.1038/s41467-018-06575-6

5 Padilla Espinosa, I. M. *et al.* Platinum nanoparticle compression: Combining in situ TEM and atomistic modeling. *Applied Physics Letters* **120** (2022). https://doi.org/10.1063/5.0078035

6 Liang, Z. *et al.* Ultimate compressive strength and severe plastic deformation of equilibrated single-crystalline copper nanoparticles. *Acta Materialia*, 120101 (2024). https://doi.org/10.1016/j.actamat.2024.120101

7 Bian, J., Yang, L., Yuan, W. & Wang, G. Influence of hydrogenation on the mechanical properties of Pd nanoparticles. *RSC advances* **11**, 3115-3124 (2021). https://doi.org/10.1039/D0RA08974E

8 Zimmerman, J. & Rabkin, E. Nanoparticle recrystallization: kinetics and size-dependent behavior. *Acta Materialia*, 121028 (2025). https://doi.org/10.1016/j.actamat.2025.121028

9 Belousov, V., Vasylyev, M., Lyashenko, L., Vilkova, N. Y. & Nieuwenhuys, B. The low-temperature reduction of Pd-doped transition metal oxide surfaces with hydrogen. *Chemical Engineering Journal* **91**, 143-150 (2003). https://doi.org/10.1016/S1385-8947(02)00147-X

10 Musket, R. Effects of contamination on the interaction of hydrogen gas with palladium: a review. *Journal of the Less Common Metals* **45**, 173-183 (1976). https://doi.org/10.1016/0022-5088(76)90265-4

11 Langhammer, C., Zhdanov, V. P., Zorić, I. & Kasemo, B. Size-Dependent Kinetics of Hydriding and Dehydriding of Pd Nanoparticles. *Physical Review Letters* **104**, 135502 (2010). https://doi.org/10.1103/PhysRevLett.104.135502

12 Alekseeva, S. *et al.* Grain-growth mediated hydrogen sorption kinetics and compensation effect in single Pd nanoparticles. *Nature Communications* **12**, 5427 (2021). https://doi.org/10.1038/s41467-021-25660-x

13 Alekseeva, S. *et al.* Grain boundary mediated hydriding phase transformations in individual polycrystalline metal nanoparticles. *Nature Communications* **8**, 1084 (2017). https://doi.org/10.1038/s41467-017-00879-9

14 Schwarz, R. B. & Khachaturyan, A. G. Thermodynamics of open two-phase systems with coherent interfaces: Application to metal-hydrogen systems. *Acta Materialia* **54**, 313-323 (2006). https://doi.org/10.1016/j.actamat.2005.08.044

15 Griessen, R., Strohfeldt, N. & Giessen, H. Thermodynamics of the hybrid interaction of hydrogen with palladium nanoparticles. *Nature Materials* **15**, 311-317 (2016). https://doi.org/10.1038/nmat4480

16 Behm, R. J., Penka, V., Cattania, M. G., Christmann, K. & Ertl, G. Evidence for ‘‘subsurface’’ hydrogen on Pd(110): An intermediate between chemisorbed and dissolved species. *The Journal of Chemical Physics* **78**, 7486-7490 (1983). https://doi.org/10.1063/1.444739

17 Grönbeck, H. & Zhdanov, V. P. Effect of lattice strain on hydrogen diffusion in Pd: A density functional theory study. *Physical Review B* **84** (2011). https://doi.org/10.1103/PhysRevB.84.052301

18 Carmiel-Kostan, M. *et al.* Nanoscale Analysis of Sulfur Poisoning Effects on Hydrogen Sorption in Single Pd Nanoparticles. *ChemRxiv* (2025). https://doi.org/10.26434/chemrxiv-2025-btqj2

19 Manchester, F., San-Martin, A. & Pitre, J. The H-Pd (hydrogen-palladium) system. *Journal of phase equilibria* **15**, 62-83 (1994). https://doi.org/10.1007/BF02667685

20 Delmelle, R. *et al.* Effect of structural defects on the hydriding kinetics of nanocrystalline Pd thin films. *International Journal of Hydrogen Energy* **40**, 7335-7347 (2015). https://doi.org/10.1016/j.ijhydene.2015.04.017
