# Supplementary material for: Hydride formation pressures and kinetics in individual Pd nanoparticles with systematically varied levels of plastic deformation
Source: Nat Commun. 2025 Oct 17;16:9242. doi: 10.1038/s41467-025-64311-3 (PMC12534434; doi:10.1038/s41467-025-64311-3)
Supplement: Supplementary file 2 — Description of Additional Supplementary Files [file 41467_2025_64311_MOESM2_ESM.pdf]

Description of Additional Supplementary Files

File Name: Supplementary Data 1
Description: The full hydrogenation history of each sample (Sample 1 and Sample 2). Every hydrogenation cycle is labelled as "Kinetics" or "Isotherm" depending on the measurement mode of that specific cycle. For kinetic cycles, the system pressure before hydrogenation, the pressure during hydrogenation, the time spent in hydrogen as well as the time for desorption (time spent pumping before the next cycle) is stated for every cycle. For isotherms, the starting and final hydrogen partial pressures as well as the total time of the isotherm is stated for every cycle.
